# Atomic-scale redox-potential-mediated engineering of 0D/2D Cu–Cu_2_O/MO_*x*_(OH)_*y*_ heterojunctions for efficient nitrate electroreduction to ammonia

**DOI:** 10.1039/d5sc08998k

**Published:** 2026-01-23

**Authors:** Tuo Zhang, Tianzhi Hao, Xiangyang Hou, Yuhui Yin, Guowen Hu, Genping Meng, Shihao Sun, Hua Li, Baodui Wang

**Affiliations:** a State Key Laboratory of Natural Product Chemistry, College of Chemistry and Chemical Engineering, Lanzhou University Lanzhou 730000 Gansu China huali@lzu.edu.cn wangbd@lzu.edu.cn; b Department of Chemistry and Biochemistry, Florida International University USA

## Abstract

The precise construction of zero-dimensional/two-dimensional (0D/2D) heterojunctions is often hindered by interfacial lattice mismatches and uncontrolled phase transitions, limiting their efficacy in electrocatalysis. Herein, we report a widely applicable redox-potential-mediated strategy for the atomically defined fabrication of 0D/2D Cu–Cu_2_O/MO_*x*_(OH)_*y*_ heterojunctions (M = Ni, Fe, Mn, Co, Cr). This approach leverages the inherent differences in standard reduction potentials between Cu and transition metals to drive selective oxidation and ultrasound-assisted hydrolysis of pre-synthesized CuM alloy nanoparticles. This process results *in situ* phase separation, forming epitaxially embedded Cu–Cu_2_O nanoparticles within ultrathin MO_*x*_(OH)_*y*_ nanosheets. As a proof of concept, the Cu–Cu_2_O/Ni(OH)_2_ heterojunction exhibits exceptional performance in the electrocatalytic nitrate reduction reaction (eNITRR), achieving an outstanding ammonia yield rate of 12,974.5 µg cm^−2^ h^−1^ (at a mass loading of 1 mg cm^−2^) and a Faradaic efficiency of 98.15%, ranking it among the high-performing catalysts reported to date. Mechanistic studies reveal a synergistic interfacial effect: Cu–Cu_2_O promotes nitrate adsorption and activation, while Ni(OH)_2_ selectively cleaves H_2_O to generate reactive *H species, thereby accelerating the hydrogenation steps. This redox-guided synthesis provides a useful framework for the atomic-scale engineering of heterointerfaces, paving the way for advanced electrocatalysts in sustainable nitrogen valorization and beyond.

## Abstract 2

A redox-potential-guided strategy is developed for the precise fabrication of 0D/2D Cu–Cu_2_O/MO_*x*_(OH)_*y*_ (M = Ni, Fe, Mn, Co, Cr) heterojunctions *via* ultrasound-assisted selective oxidation and hydrolysis.

## Graphical abstract

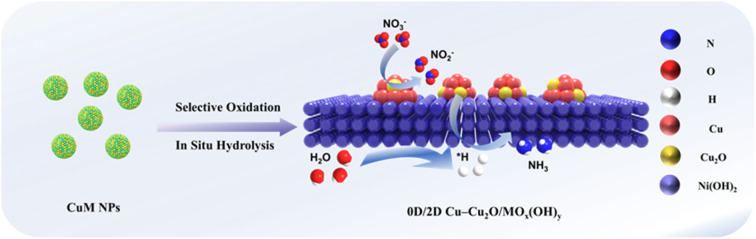

## Introduction

The rational design and precise synthesis of hetero-nanoarchitectures from structurally dissimilar building blocks represent a foundational challenge in materials chemistry.^1^ Integrating zero-dimensional nanoparticles (0D NPs) with two-dimensional nanosheets (2D NSs) is a particularly compelling strategy, as such 0D/2D heterojunctions can combine the high surface activity of NPs with the exceptional charge transport and confinement effects of 2D matrices, often leading to emergent catalytic properties.^2–4^ However, the bottom-up construction of these hybrid materials with atomic-level interfacial control is often hindered by lattice mismatches and uncontrolled phase transformations during synthesis, resulting in poorly defined interfaces that obscure fundamental structure–property relationships and limit catalytic performance.^5,6^

This synthetic challenge is acutely evident in the development of advanced electrocatalysts for complex, multi-step reactions such as the electrochemical nitrate reduction reaction (eNITRR). The conversion of nitrate (NO_3_^−^) pollutants to valuable ammonia (NH_3_) presents a sustainable route for simultaneous wastewater remediation and chemical production.^7–9^ Copper-based materials have shown exceptional promise for eNITRR, primarily due to the favorable alignment of their d-orbitals with nitrate orbitals, enabling strong adsorption and facile activation of NO_3_^−^.^9,10^ Recent advances highlight that constructing Cu/Cu_2_O heterointerfaces can further enhance performance by synergistically modulating the d-band center to optimize the adsorption energies of key intermediates.^11–13^ Nevertheless, the complete conversion of NO_3_^−^ to NH_3_ involves a complex, multi-step hydrogenation pathway that requires a continuous and efficient supply of active hydrogen atoms (*H), typically sourced from water dissociation.^14–18^ Conventional Cu-based catalysts, while effective for nitrate activation, generally exhibit limited efficiency in cleaving the H–OH bond, creating a critical bottleneck in the proton-coupled electron transfer processes and ultimately restricting the overall NH_3_ production rate.^7,8,19^

A potential solution lies in the strategic design of heterojunctions that couple distinct active sites, one for nitrate adsorption/activation and another for efficient water dissociation and *H generation. In this context, 2D transition metal hydroxides (MO_*x*_(OH)_*y*_) have recently emerged as exceptional platforms for activating water molecules and generating *H species.^20–23^ Their ultrathin nature provides abundant unsaturated coordination sites and facilitates rapid interfacial charge transfer. Consequently, the integration of 0D Cu-based NPs with 2D MO_*x*_(OH)_*y*_ NSs is a highly promising avenue for creating synergistic catalysts. However, the realization of such precisely controlled 0D/2D architectures remains a formidable synthetic challenge. Conventional methods often fail to achieve epitaxial integration or control interfacial redox chemistry, leading to incoherent interfaces and suboptimal catalytic synergy.^24,25^

Herein, we propose and demonstrate that the inherent standard reduction potentials (E^0^) of constituent metals can serve as a powerful, predictive descriptor to guide the precise synthesis of 0D/2D heterojunctions. We present a general redox-potential-mediated strategy for fabricating a class of atomically defined Cu–Cu_2_O/MO_*x*_(OH)_*y*_ (M = Ni, Fe, Mn, Co, Cr) heterostructures. The core of our approach lies in exploiting the thermodynamic disparity in standard reduction potentials between copper (E^0^(Cu^2+^/Cu^0^) = +0.34 V) and various first-row transition metals, including nickel (E^0^(Ni^2+^/Ni^0^) = −0.25 V), iron (E^0^(Fe^2+^/Fe^0^) = −0.44 V), manganese (E^0^(Mn^2+^/Mn^0^) = −1.18 V), cobalt (E^0^(Co^2+^/Co^0^) = −0.28 V), and chromium (E^0^(Cr^3+^/Cr^0^) = −0.74 V). Starting from pre-synthesized CuM alloy NPs, controlled oxidation and ultrasound-assisted hydrolysis selectively leach and transform the more oxidizable metal M into ultrathin MO_*x*_(OH)_*y*_ NSs, while Cu undergoes controlled oxidation to form epitaxially embedded Cu–Cu_2_O NPs. As a compelling proof-of-concept, the optimal Cu–Cu_2_O/Ni(OH)_2_ heterojunction exhibits an exceptional Faradaic efficiency of 98.15% and a record-high ammonia yield rate of 12,974.5 µg cm^−2^ h^−1^. Through a combination of *in situ* spectroscopic techniques and density functional theory (DFT) calculations, we decipher the interfacial synergy: Cu–Cu_2_O acts as the primary site for nitrate adsorption and activation, while Ni(OH)_2_ efficiently dissociates water to supply *H species, collectively accelerating the hydrogenation steps. This work establishes a widely applicable paradigm for using fundamental electrochemical properties to navigate the precision synthesis of complex functional materials, with implications that extend well beyond electrocatalysis.

## Results and discussion

### Synthesis and structural characterization

**Fig. 1:**
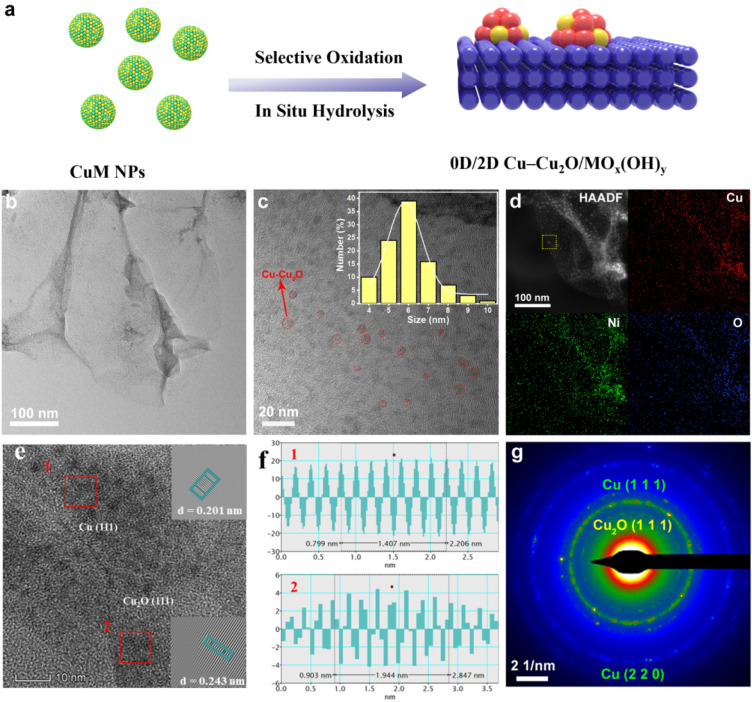
Synthesis and morphological characterization of Cu–Cu_2_O/Ni(OH)_2_. (a) Schematic illustration of the redox-potential-mediated synthesis of Cu–Cu_2_O/MO_*x*_(OH)_*y*_ heterojunctions. (b) TEM image of the as-synthesized Cu–Cu_2_O/Ni(OH)_2_. (c) High-resolution TEM (HR-TEM) image and the corresponding particle size distribution of Cu–Cu_2_O NPs. (d) HAADF-STEM image and corresponding elemental mapping for Cu, Ni, and O. (e) HR-TEM image showing lattice fringes of different crystalline domains; insets are magnified FFT-filtered images of regions 1 and 2. (f) Corresponding lattice spacing analysis of regions 1 and 2 marked in (e). (g) SAED pattern of Cu–Cu_2_O/Ni(OH)_2_.

The Cu–Cu_2_O/MO_*x*_(OH)_*y*_ heterojunction was synthesized *via* a three-step redox-potential-mediated process, which capitalizes on the distinct standard reduction potentials of Cu and transition metal M to achieve selective oxidation and phase separation. As illustrated in Fig. 1a, the process involves: (1) liquid-phase reduction of Cu-oleate and M-oleate complexes using sodium borohydride at ambient temperature to form CuM alloy nanoparticles (NPs); (2) selective oxidation of the as-synthesized CuM NPs under controlled conditions; and (3) ultrasound-assisted hydrolysis to promote the *in situ* growth of two-dimensional MO_*x*_(OH)_*y*_ nanosheets (NSs). The resulting CuM NPs were characterized by typical transmission electron microscopy (TEM) (Fig. S1, SI), X-ray powder diffraction (XRD) (Fig. S2, SI). The ultrasound treatment not only provides the energy for hydrolysis and exfoliation but also enhances mass transfer, facilitating the formation of ultrathin NSs. Specifically, when CuNi NPs are subjected to ultrasound in deionized water, the Ni component (E^0^(Ni^2+^/Ni^0^) = −0.25 V) is preferentially oxidized and hydrolyzed to form ultrathin Ni(OH)_2_ NSs (Fig. 1b), while the Cu (E^0^(Cu^2+^/Cu^0^) = +0.34 V) undergoes partial oxidation to form Cu–Cu_2_O NPs anchored on the Ni(OH)_2_ substrate (Fig. 1c). TEM analysis confirms the two-dimensional sheet-like morphology of Ni(OH)_2_ and the homogeneous dispersion of Cu–Cu_2_O NPs with an average size of 5.9 nm on the NSs. The formation of heterojunction interfaces between Cu and Cu_2_O NPs is also clearly observed (Fig. S3, SI). Elemental mapping *via* high-angle annular dark-field scanning transmission electron microscopy (HAADF-STEM) (Fig. 1d) confirms the uniform distribution of Cu, Ni, and O throughout the structure. High-resolution TEM (HR-TEM) images (Fig. 1e and f) reveal lattice fringes corresponding to Cu (111) (0.201 nm) and Cu_2_O (111) (0.243 nm), with insets showing magnified FFT-filtered images of regions 1 and 2. The selected-area electron diffraction (SAED) pattern (Fig. 1g) further confirms the coexistence of Cu and Cu_2_O crystalline phases, providing clear evidence for the successful construction of the Cu–Cu_2_O/Ni(OH)_2_ heterojunction *via* the redox-mediated strategy.

### Crystalline phase and valence state analysis

**Fig. 2:**
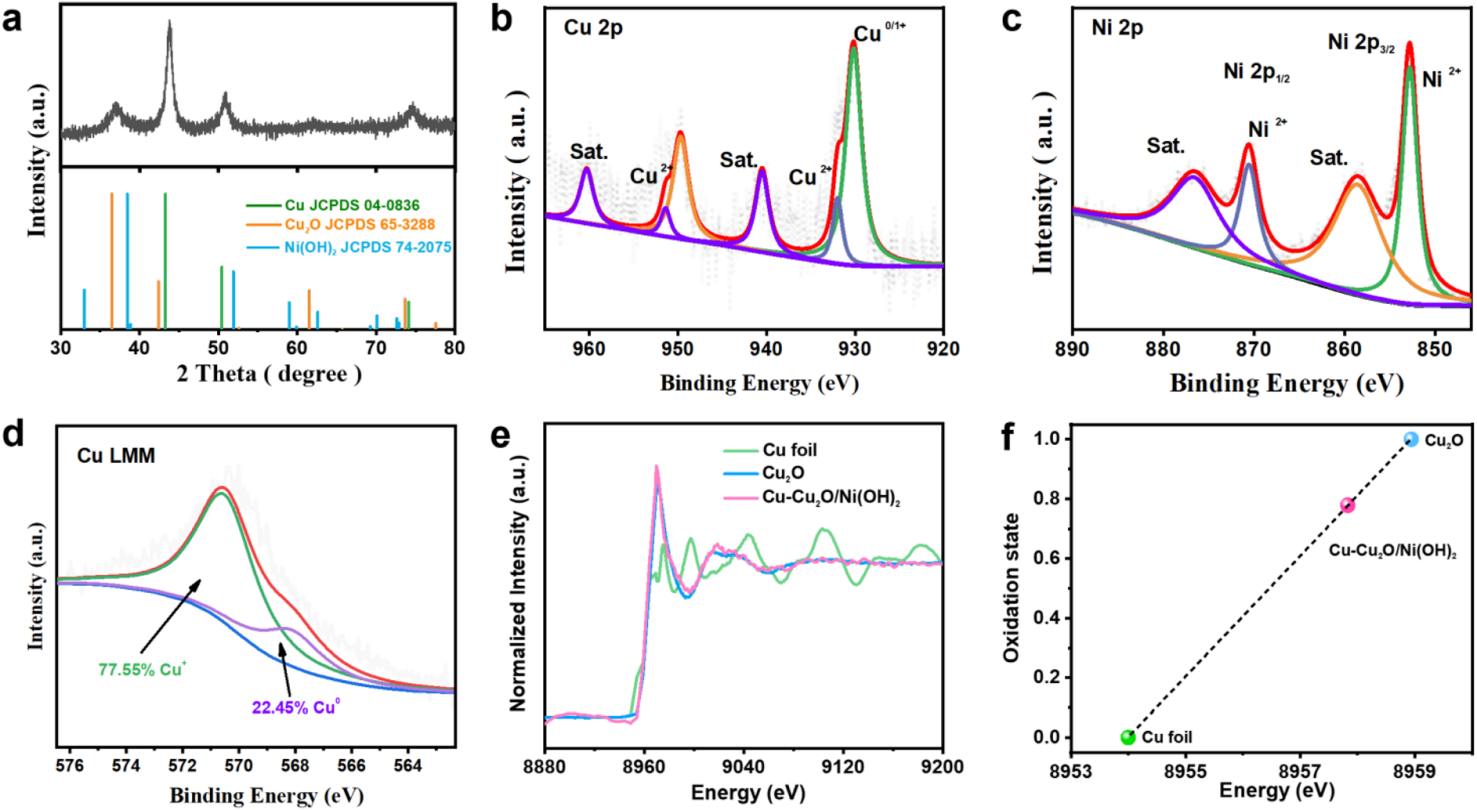
Crystalline phase and valence state of Cu–Cu_2_O/Ni(OH)_2_. (a) XRD pattern of Cu–Cu_2_O/Ni(OH)_2_. (b) High-resolution XPS spectrum of Cu 2p. (c) High-resolution XPS spectrum of Ni 2p. (d) Cu LMM Auger electron spectrum. (e) Cu K-edge XANES spectra of Cu foil, Cu_2_O, and Cu–Cu_2_O/Ni(OH)_2_. (f) Correlation between absorption edge energy and the oxidation state of Cu species.

XRD analysis reveals the coexistence of metallic Cu and Cu_2_O crystalline phases in the synthesized material (Fig. 2a), validating the spontaneous growth of Cu–Cu_2_O NPs on Ni(OH)_2_ nanosheets. The diffraction peaks at 43.47° and 50.37° are assigned to the (111) and (200) planes of cubic Cu (JCPDS No. 04-0836), while peaks at 29.54°, 36.42°, 42.30°, and 61.34° correspond to the (110), (111), (200), and (220) planes of cubic Cu_2_O (JCPDS No. 65-3288).^26^ The absence of discernible diffraction peaks for Ni(OH)_2_ suggests its amorphous nature.^27^ The surface chemical states of the Cu–Cu_2_O/Ni(OH)_2_ heterojunction were determined by XPS. The Cu 2p spectrum (Fig. 2b) exhibited peaks at binding energies of 930.47 eV and 950.27 eV, indicative of Cu^0^/^1+^ species.^28–30^ The peak at ∼932.31 eV can be attributed to residual surface copper-oleate species. In the Ni 2p spectrum (Fig. 2c), the peaks at 852.68 eV and 871.94 eV are characteristic of Ni^2+^ species,^31^ with satellite peaks observed at higher binding energies.^32,33^ Auger electron spectroscopy (AES) analysis provided further insight into the copper oxidation states. The peaks at 568 eV and 570.5 eV corresponded to Cu^0^ and Cu^+^, with relative abundances of 22.45% and 77.55%, respectively (Fig. 2d), indicating that copper predominantly exists in the +1 oxidation state.^34^ Cu K-edge XANES analysis (Fig. 2e) corroborated these findings. The absorption edge position and spectral shape closely resembled that of the Cu_2_O reference, indicating Cu^+^ as the predominant species. A linear correlation between absorption edge energy and formal valence state (Fig. 2f) yielded an average Cu oxidation state of approximately +0.78, consistent with the XPS and AES results. In summary, combined characterization by XRD, XPS, AES, and XANES consistently demonstrates that copper in the composite is primarily in the Cu^+^ state, with a minor contribution from Cu^0^, a distribution favorable for interfacial electron transfer and catalytic selectivity.

### Formation pathway and structural evolution

**Fig. 3:**
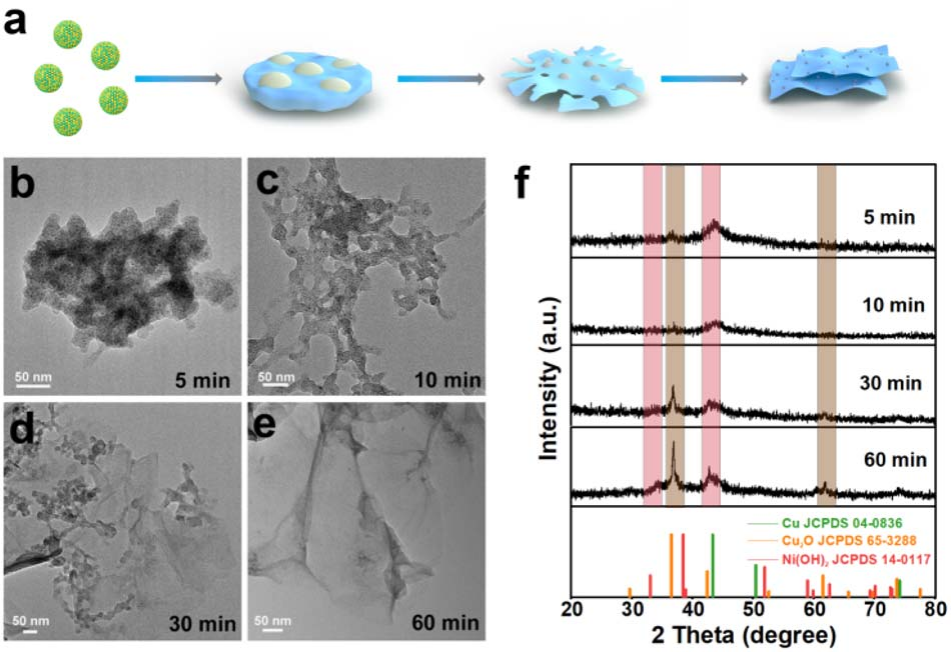
Formation pathway and structural evolution of Cu–Cu_2_O/Ni(OH)_2_. (a) Schematic illustration of the formation process. (b–e) TEM images of CuNi nanoparticles subjected to ultrasound for different durations. (f) XRD patterns tracking the phase transformation during ultrasonic treatment.

To elucidate the formation mechanism of the Cu–Cu_2_O/Ni(OH)_2_ heterojunction, we monitored the structural evolution during ultrasonic treatment (Fig. 3a). TEM images (Fig. 3b–e and S4, SI) and time-dependent XRD spectra (Fig. 3f and S5, SI) track the transformation. Initially, Ni within the CuNi alloy NPs is preferentially oxidized and hydrolyzed to form Ni(OH)_2_ NSs, which become thinner with prolonged ultrasonication. Concurrently, Cu is oxidized to form Cu–Cu_2_O NPs that deposit onto the growing NSs. The XRD peaks corresponding to the CuNi alloy (*e.g.*, at 43.1°) diminish, while those for Cu (43.3°) and Cu_2_O (36.42°, 61.52°) emerge and intensify. No metallic Ni peaks are observed, confirming the complete hydrolysis of Ni. Both TEM and XRD confirm the redox-driven phase separation, resulting in well-defined 0D/2D heterojunctions. The relative content of copper and nickel remains stable throughout the process (Table S1), indicating a conservative transformation.

### Extension to other metal systems: widely applicable synthesis strategy

To demonstrate the wide applicability of our redox-potential-mediated strategy, we synthesized Cu–Cu_2_O/MO_*x*_(OH)_*y*_ heterojunctions through the simple introduction of the corresponding as-synthesized CuM NPs (M = Cr, Mn, Fe, and Co). XRD analysis (Fig. S6, SI) confirmed the coexistence of Cu and Cu_2_O phases in all cases. XPS (Fig. S7, SI), AES (Fig. S8, SI), and M 2p spectra (Fig. S9, SI) indicated that the introduction of different M elements had a minimal impact on the oxidation state of Cu, further validating the robustness of our method. The hydrolysis of metal M led to the formation of the respective MO_*x*_(OH)_*y*_, as evidenced by their corresponding lattice fringes (Fig. S10, SI).

**Fig. 4:**
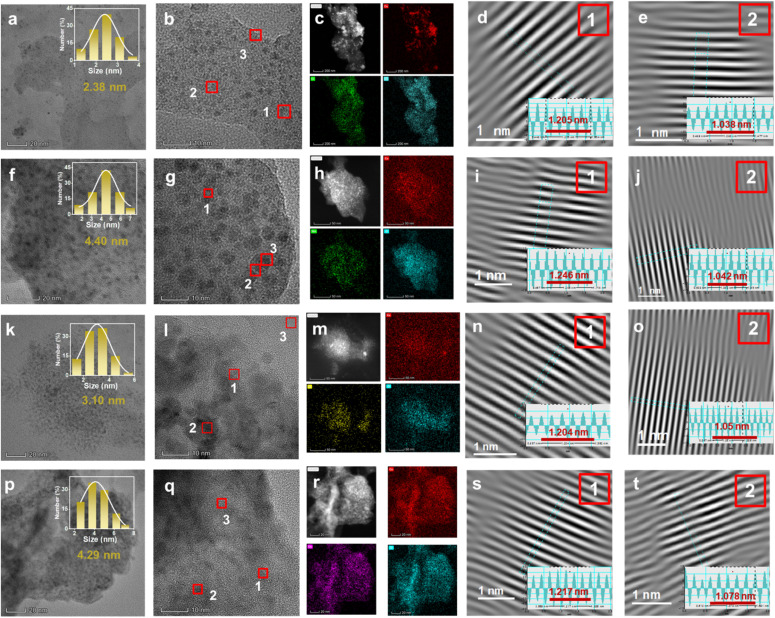
TEM images of four different materials, arranged side by side, provide multilayered information for each material. (a) Low-magnification TEM image, (b) HR-TEM image, (c) HAADF-STEM image and corresponding elemental mapping, (d) Cu_2_O (111) crystal plane at position 1 with a lattice spacing of 0.241 nm, and (e) Cu (111) crystal plane at position 2 with a lattice spacing of 0.208 nm, for Cu–Cu_2_O/CrOOH. (f) Low-magnification TEM image, (g) HR-TEM image, (h) HAADF-STEM image and corresponding elemental mapping, (i) Cu_2_O (111) crystal plane at position 1 with a lattice spacing of 0.249 nm, and (j) Cu (111) crystal plane at position 2 with a lattice spacing of 0.208 nm, for Cu–Cu_2_O/Mn(OH)_2_. (k) Low-magnification TEM image, (l) HR-TEM image, (m) HAADF-STEM image and corresponding elemental mapping, (n) Cu_2_O (111) crystal plane at position 1 with a lattice spacing of 0.241 nm, and (o) Cu (111) crystal plane at position 2 with a lattice spacing of 0.210 nm, for Cu–Cu_2_O/FeOOH. (p) Low-magnification TEM image, (q) HR-TEM image, (r) HAADF-STEM image and corresponding elemental mapping, (s) Cu_2_O (111) crystal plane at position 1 with a lattice spacing of 0.243 nm, and (t) Cu (111) crystal plane at position 2 with a lattice spacing of 0.216 nm, for Cu–Cu_2_O/Co(OH)_2_. The second column highlights regions 1, 2, and 3 for detailed crystal analysis, corresponding to the Cu_2_O (111) crystal plane, Cu (111) crystal plane, and MO_*x*_(OH)_*y*_, respectively.

TEM analysis (Fig. 4) revealed the particle size distribution of Cu–Cu_2_O NPs grown on various MO_*x*_(OH)_*y*_ NSs. Cu–Cu_2_O/CrOOH exhibited the smallest Cu–Cu_2_O particle size (2.38 nm), followed by Cu–Cu_2_O/FeOOH (3.10 nm), Cu–Cu_2_O/Co(OH)_2_ (4.29 nm), and Cu–Cu_2_O/Mn(OH)_2_ (4.40 nm). This trend suggests that CrOOH most effectively restricts copper particle nucleation and growth, while Mn(OH)_2_ and Co(OH)_2_ exert a weaker influence. EDS mapping confirmed the uniform distribution of Cr, Mn, Fe, and Co elements in their respective samples. HR-TEM analysis revealed variations in lattice spacings: Cu_2_O(111) spacings were similar for Cu–Cu_2_O/CrOOH and Cu–Cu_2_O/FeOOH (∼0.241 nm), while Cu–Cu_2_O/Mn(OH)_2_ exhibited the largest spacing (0.249 nm). For Cu(111), spacings ranged from 0.208 nm (Cu–Cu_2_O/CrOOH, Cu–Cu_2_O/Mn(OH)_2_) to 0.216 nm (Cu–Cu_2_O/Co(OH)_2_). These results underscore the crucial role of the M element and ultrasound in regulating the final structure of the Cu-based heterojunction catalysts, highlighting the method's versatility.

### Electrocatalytic nitrate reduction performance

The electrochemical nitrate reduction reaction (eNITRR) performance of the catalysts and carbon felt was evaluated in a standard three-electrode H-type cell. Control samples, including Ni(OH)_2_ NSs, Cu–Cu_2_O NPs, and a physical mixture of Cu–Cu_2_O + Ni(OH)_2_, were synthesized and characterized (Fig. S11, S12, SI). Product concentrations (NO_3_^−^, NO_2_^−^, NH_3_) were quantified using colorimetric methods (Fig. S13–S15) and ion chromatography (IC) (Fig. S16, S17, SI).

**Fig. 5:**
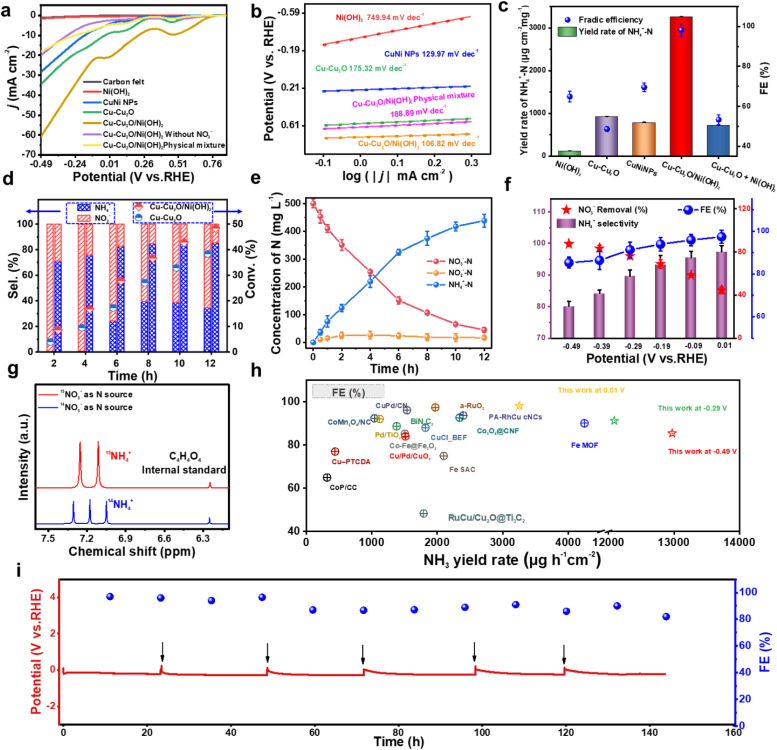
eNITRR performance of different electrocatalyst. (a) *j*-V plots of electrocatalytic reduction of nitrate over carbon felt, Ni(OH)_2_, CuNi NPs, Cu–Cu_2_O, Cu–Cu_2_O + Ni(OH)_2_ and Cu–Cu_2_O/Ni(OH)_2_ heterojunctions (80% iR corrected). (b) Tafel slopes for Ni(OH)_2_, CuNi NPs, Cu–Cu_2_O, Cu–Cu_2_O + Ni(OH)_2_ and Cu–Cu_2_O/Ni(OH)_2_ catalysts. (c) Faradaic efficiency and yield of NH_4_^+^-N over Ni(OH)_2_, CuNi NPs, Cu–Cu_2_O, Cu–Cu_2_O + Ni(OH)_2_ and Cu–Cu_2_O/Ni(OH)_2_ catalysts. (d) Mass-normalized selectivity of NO_2_^−^ and NH_4_^+^ over Cu–Cu_2_O and Cu–Cu_2_O/Ni(OH)_2_ catalysts at 0.01 V (*vs.*RHE),1 g L^−1^ NO_3_^−^-N as a function of reaction time. (e) NO_3_^−^-N consumption, NO_2_^−^-N generation, and NH_4_^+^-N generation over Cu–Cu_2_O/Ni(OH)_2_ catalysts at different times with 500 mg L^−1^ NO_3_^−^-N. (f) NH_4_^+^ selectivity, NO_3_^−^ removal rate and Faradaic efficiency of Cu–Cu_2_O/Ni(OH)_2_ catalysts at different potentials. (g) Detection of ^14^NH_4_^+^ and isotopically labeled ^15^NH_4_^+^ by ^1^H NMR. (h) Comparison of the eNITRR performance of the Cu–Cu_2_O/Ni(OH)_2_ with other catalysts reported in the literature. (i) The long-term electrocatalytic stability of eNITRR on Cu–Cu_2_O/Ni(OH)_2_ was evaluated in an H-type cell at a current density of 20 mA cm^−2^. The black arrows indicate the replenishment of fresh electrolytes.

For each sample, linear sweep voltammetry (LSV) curves were acquired to confirm the current density (*j*) of the reaction (Fig. 5a). The LSV curves were recorded after 5 cycles sweeping between the potential from −0.49 V to 1.01 V (*vs.* RHE). As shown in Fig. 5a, in the presence of NO_3_^−^, with the exception of carbon felt and Ni(OH)_2_ NSs, the *j* of other catalysts significantly increases as the reduction overpotential increases. At the same potential, the Cu–Cu_2_O/Ni(OH)_2_ catalyst exhibits the highest *j*, indicating its superior electrocatalytic nitrate reduction activity. Fig. 5b shows the Tafel slope plots for Ni(OH)_2_, CuNi NPs, Cu–Cu_2_O, Cu–Cu_2_O + Ni(OH)_2_, and Cu–Cu_2_O/Ni(OH)_2_, where Cu–Cu_2_O/Ni(OH)_2_ demonstrates a Tafel slope of 106.82 mV dec^−1^, signifying its lowest overpotential and more favorable kinetics for the eNITRR. The Cu–Cu_2_O/Ni(OH)_2_ heterojunction exhibits the smallest semicircle diameter in the Nyquist plot (Fig. S22a, SI), indicating the lowest charge-transfer resistance among the three catalysts and thus the most favorable reaction kinetics. Fig. S22c (SI) shows that the local pH at the Cu–Cu_2_O/Ni(OH)_2_ electrode surface gradually increases during the reaction, indicating interfacial alkalization caused by continuous proton consumption in electrochemical nitrate reduction. In Fig. 5c, a Faradaic efficiency (FE) comparison is presented for Ni(OH)_2_, CuNi NPs, Cu–Cu_2_O, Cu–Cu_2_O + Ni(OH)_2_, and Cu–Cu_2_O/Ni(OH)_2_ catalysts in the NH_3_ production process. The FE of Cu–Cu_2_O/Ni(OH)_2_ is 98.15%, significantly surpassing that of Ni(OH)_2_ (64.85%), CuNi NPs (69.50%), Cu–Cu_2_O (48.58%) and Cu–Cu_2_O + Ni(OH)_2_ (53.30%). Additionally, the NH_3_ yield of Cu–Cu_2_O/Ni(OH)_2_ is notably higher than that of Ni(OH)_2_ and Cu–Cu_2_O. Fig. 5d shows the change of mass normalized selectivity of NO_2_^−^ and NH_4_^+^ on Cu–Cu_2_O and Cu–Cu_2_O/Ni(OH)_2_ over time at a low overpotential (0.01 V *vs.* RHE). During the reaction, Cu–Cu_2_O generates a significant amount of harmful intermediate NO_2_^−^ products. However, on the Cu–Cu_2_O/Ni(OH)_2_ catalyst, the selectivity of NO_2_^−^ decreases over time while the selectivity of NH_3_ increases. After 24 h, the selectivity of NH_3_ reaches 98.5%, whereas the selectivity of NO_2_^−^ is only 1.5%. The outstanding eNITRR performance of Cu–Cu_2_O/Ni(OH)_2_ can be attributed to the synergistic effect at the interface of Cu–Cu_2_O NPs and 2D Ni(OH)_2_ NSs, in which NO_3_^−^ is rapidly converted to NO_2_^−^ by Cu–Cu_2_O NPs and subsequently reduced to NH_4_^+^ by *H produced from activated water in Ni(OH)_2_ NSs. Fig. 5e shows the change of NO_3_^−^–N, NH_4_^+^-N, and NO_2_^−^-N concentrations with reaction time at a working potential of −0.49 V, and the initial NO_3_^−^-N concentration is set at 500 mg L^−1^. As the reaction time increases, the concentration of NO_3_^−^-N continues to decrease while that of NH_4_^+^–N continues to increase; however, the concentration level remains relatively low for NO_2_^−^N, indicating the remarkable selectivity of the Cu–Cu_2_O/Ni(OH)_2_ catalyst in reducing NO_3_^−^ to NH_4_^+^ with minimal generation of the byproduct NO_2_^−^. The electrochemical active surface area (ECSA) of different materials was also tested, and the experimental results show that Cu–Cu_2_O/Ni(OH)_2_ has the highest ECSA, suggesting it has the largest contact area with reactants, as shown in Fig. S18 (SI).

In order to further investigate the catalytic performance of the Cu–Cu_2_O/Ni(OH)_2_ catalyst, the removal rate of NO_3_^−^, selectivity for NH_4_^+^, and FE were measured at different potentials. As depicted in Fig. 5f, both NH_4_^+^ selectivity and FE gradually decrease with a reduction in potential due to increased generation of NO_2_^−^ and competitive HER at lower potentials. Conversely, the NO_3_^−^ removal rate increases gradually with decreasing potential as more energy is utilized for the electrocatalytic nitrate reduction process at low potential. Furthermore, the catalytic performance of Cu–Cu_2_O/Ni(OH)_2_ under different pH conditions (Fig. S22b, SI) shows that both the NH_3_ yield and Faradaic efficiency (FE) initially increase and then decrease with increasing electrolyte pH, indicating that moderately alkaline conditions favor nitrate reduction, whereas excessive alkalinity may limit proton availability. In addition, as shown in Fig. S22d (SI), the NH_3_ yield increases with nitrate concentration, while the FE exhibits a volcano-type dependence, suggesting that although higher nitrate concentrations enhance reaction rates, mass-transport limitations reduce electron utilization efficiency at high concentrations. The ammonia origin was determined through ^15^N isotope labeling experiments. As shown in Fig. 5g and S19 (SI), utilization of Na^14^NO_3_ as the nitrogen source in the electrolyte resulted in three characteristic ^14^NH_4_^+^ peaks at chemical shifts of 6.91 ppm, 7.04 ppm, and 7.17 ppm in the post-reaction electrolyte's ^1^H NMR spectrum. Conversely, when Na^15^NO_3_ was employed as the nitrate source, the resulting ^1^H NMR spectra exhibited distinct double peaks corresponding to ^15^NH_4_^+^ at chemical shifts of 6.95 ppm and 7.13 ppm with no additional peaks present, confirming that the obtained ^15^NH_4_^+^ originated solely from electrochemical reduction of Na^15^NO_3_ in presence of catalyst rather than environmental contamination.^35^ In comparison to the majority of reported electrocatalytic nitrate reduction catalysts, the Cu–Cu_2_O/Ni(OH)_2_ catalyst exhibits superior NH_3_ production rate and Faradaic efficiency at different potentials (Fig. 5h and Table S2). Furthermore, Cu–Cu_2_O/Ni(OH)_2_ exhibited excellent electrochemical stability, maintaining long-term stability for over 140 hours at a current density of 20 mA cm^−2^, with negligible decay in activity and Faradaic efficiency (Fig. 5i and S20, SI). This demonstrates the strong application potential of Cu–Cu_2_O/Ni(OH)_2_ in electrolytic systems.^36^ After XRD, TEM, and XPS characterization analyses (Fig. S23, SI), a gradual conversion of partial Cu_2_O to metallic Cu was observed in Cu–Cu_2_O/Ni(OH)_2_ during the cyclic electrocatalytic process.^37^ In addition, during the catalytic process, the morphology of the Cu–Cu_2_O/Ni(OH)_2_ catalyst did not undergo any significant changes. This indicates that the Cu–Cu_2_O/Ni(OH)_2_ catalyst can maintain its structural and morphological stability during the reaction process, ensuring the continuity and reliability of its catalytic activity.

### Mechanistic insights from synergistic interface effects

**Fig. 6:**
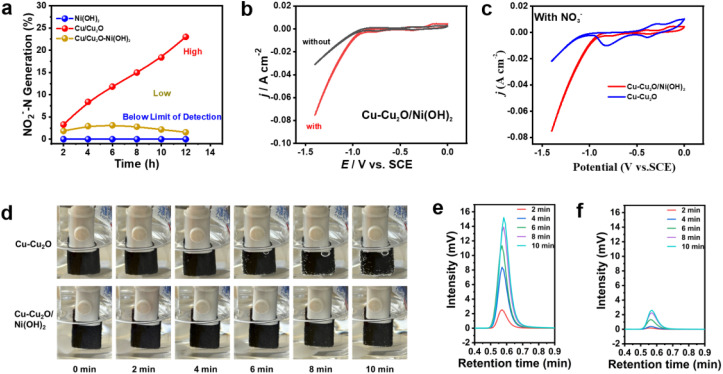
Electrochemical methods for mechanistic investigation. (a) NO_2_^−^ generation as a function of time over Ni(OH)_2_ (Below Limit of detection), Cu–Cu_2_O (High) and Cu–Cu_2_O/Ni(OH)_2_ (Low) (b) CV measurements of Cu/Cu_2_O–Ni(OH)_2_ with (red line) and without (black line) nitrate in 0.1 M NaOH solution and 500 mg L^−1^ NO_3_^−^-N with 100 mV s^−1^ sweeping speed. (c) CV measurements of Cu–Cu_2_O and Cu/Cu_2_O–Ni(OH)_2_ in 500 mg L^−1^ NO_3_^−^-N solution with 100 mV s^−1^ sweeping speed. The CV curves were recorded after 5 cycles of surface cleaning. (d) Digital photographs of hydrogen precipitation from cathodes in the presence of NO_3_^−^ at a potential of −0.49 V (*vs.*RHE). (e) The Cu–Cu_2_O and (f) Cu–Cu_2_O/Ni(OH)_2_ catalyzed H_2_ generation curves in the presence of NO_3_^−^.

To further elucidate the mechanism of Cu–Cu_2_O/Ni(OH)_2_ electrocatalytic nitrate reduction, a series of electrochemical tests were conducted. Fig. 6a shows the time-dependent production of NO_2_^−^ by Ni(OH)_2_, Cu–Cu_2_O and Cu–Cu_2_O/Ni(OH)_2_. It is evident that Cu–Cu_2_O catalysis yields the highest amount of NO_2_^−^, while Ni(OH)_2_ catalysis shows minimal production. In the case of Cu–Cu_2_O/Ni(OH)_2_, only a small quantity of NO_2_^−^ is generated initially, followed by a gradual decrease over time. The cyclic voltammetry (CV) curves of Cu–Cu_2_O/Ni(OH)_2_ in the solution with and without NO_3_^−^ are compared in Fig. 6b. The presence of NO_3_^−^ induces conspicuous enhancement of cathodic current density beginning at ∼0.01 V (*vs.* RHE) (red curve), confirming the reduction activity of NO_3_^−^ at Cu–Cu_2_O/Ni(OH)_2_. As a poignant example, the CVs of Cu–Cu_2_O and Cu–Cu_2_O/Ni(OH)_2_ in NO_3_^−^ solution were acquired, shown in Fig. 6c. A clear difference is observed that the cathodic current density rises more quickly at Cu–Cu_2_O/Ni(OH)_2_ than at Cu–Cu_2_O after ∼0.01 V (*vs.* RHE), highlighting the outstanding catalytic capability of Cu–Cu_2_O/Ni(OH)_2_ on NO_3_^−^ reduction under the studying conditions.^38^ These control experiments suggest that the introduction of Ni(OH)_2_ to the catalyst system remarkably promotes its catalytic performance. The rapid consumption of *H by NO_2_^−^ is beneficial to improve FE, otherwise, the accumulation of *H is prone to forming hydrogen gas.^39^ The existing study pointed out that under the HER process at low overpotential (close to equilibrium potential), the reaction rates of HER and hydrogen oxidation reaction (HOR) are nearly comparable (*i*_HER_ ≈ *i*_HOR_) so that the contribution from the reverse path is non-negligible. Additionally, if the generated H_2_ is locally trapped inside the nano-structure of our catalyst, a worse result would be foreseen since it will suppress the HER process (*i.e.* the generation of *H). In either scenario, lower FE will be obtained.^40–43^

To more intuitively support the above hypothesis, we tracked the changes in the hydrogen generation effect of Cu–Cu_2_O/Ni(OH)_2_ and Cu–Cu_2_O in the presence of NO_3_^−^ over time using digital photos. As shown in Fig. 6d and e, for Cu–Cu_2_O, visible bubbles can be seen within 4 min, while for Cu–Cu_2_O/Ni(OH)_2_, the same hydrogen generation effect as Cu–Cu_2_O was observed more than 10 min later (Fig. 6d–f), indicating that the *H generated by Ni(OH)_2_ activated H_2_O was consumed by NO_2_^−^ generated by Cu–Cu_2_O catalyzed NO_3_^−^. It is preliminarily speculated that Cu–Cu_2_O catalyzes the reduction of NO_3_^−^ to NO_2_^−^, while Ni(OH)_2_ catalyzes water to produce *H to further promote the hydrogenation reduction of NO_2_^−^.

**Fig. 7:**
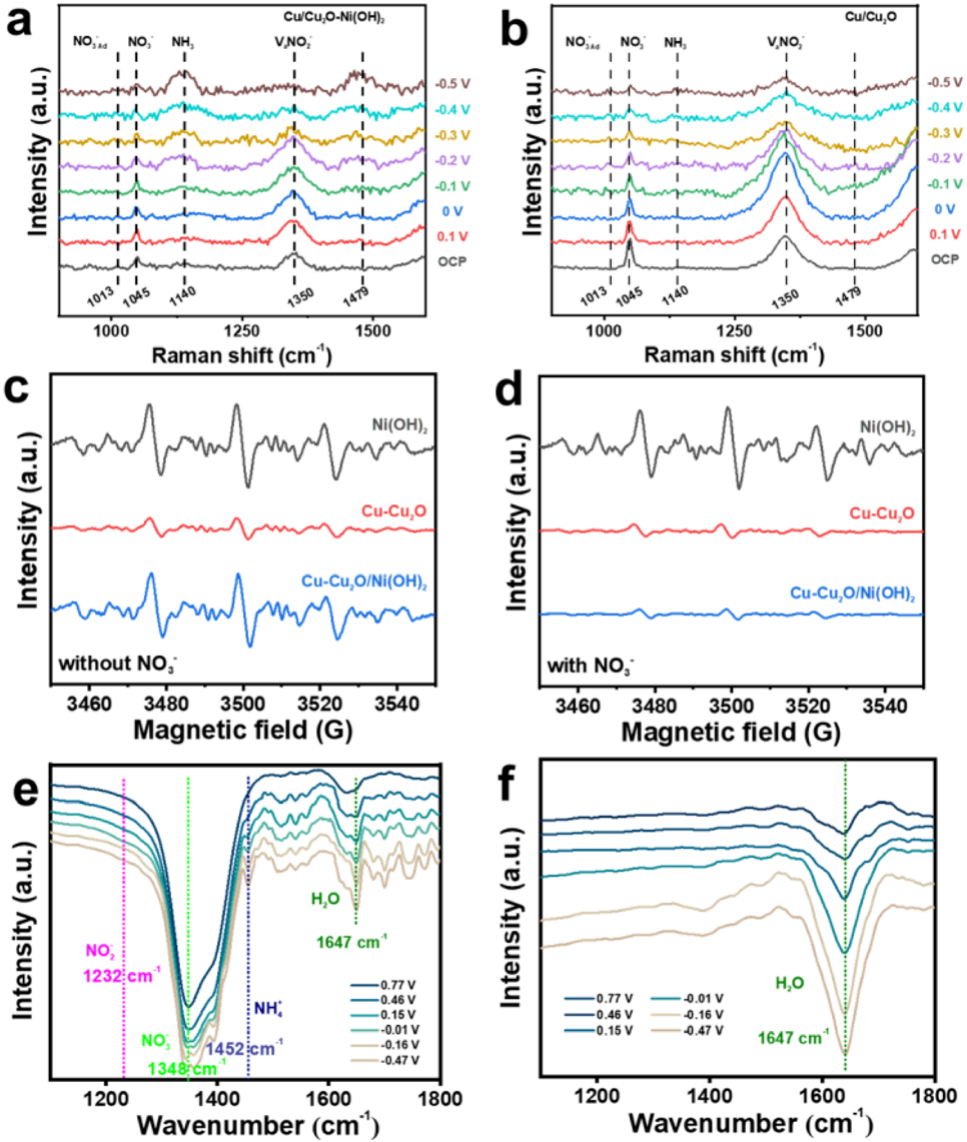
*In situ* Measurements and EPR spectra of the electrocatalyst during the reaction. (a) *In situ* Raman spectra of Cu–Cu_2_O/Ni(OH)_2_ (a)and Cu–Cu_2_O (b) during eNITRR at different potentials (V *vs.* RHE) in a mixed solution (0.1 M NaNO_3_ + 0.1 M NaOH). EPR spectra of different materials without (c) and with (d) NO_3_^−^. *In situ* infrared spectra of Cu–Cu_2_O/Ni(OH)_2_ with (e) and without (f) NO_3_^−^ at different potentials.

*In situ* electrochemical Raman spectroscopy was used to investigate the process of reducing NO_3_^−^ to NH_3_ over Cu–Cu_2_O/Ni(OH)_2_ (Fig. 7a) and Cu–Cu_2_O (Fig. 7b) catalysts in a mixed solution (0.1 M NaNO_3_ + 0.1 M NaOH) within the potential range of 0.1 to −0.5 V (*vs.* RHE). At 0.1V, the Raman band at 1045 cm^−1^ was clearly observed on both Cu–Cu_2_O/Ni(OH)_2_ and Cu–Cu_2_O catalysts, which can be attributed to the vibration mode of aqueous NO_3_^−^.^44^ As the potential decreases and nitrate is continuously consumed in the aqueous solution, the intensity of the Raman band at 1045 cm^−1^ gradually diminishes. Correspondingly, the Raman band of adsorbed NO_3_^−^ (NO_3_^−^_Ad_) appears and gradually strengthens at 1013 cm^−1^. At 0.1 V (*vs.* RHE), the Raman band of 1350 cm^−1^ is attributed to the stretching vibrations of NO_2_^−^,^45^ and its intensity decreases with decreasing potential. It is evident that the reduction degree of the Raman band of NO_2_^−^ in Cu–Cu_2_O/Ni(OH)_2_ is significantly higher than that in Cu–Cu_2_O, indicating that Cu–Cu_2_O/Ni(OH)_2_ can greatly enhance water activation and promote the progressive hydrogenation process from NO_2_^−^ to NH_3_. The two Raman bands at 1140 cm^−1^ and 1479 cm^−1^ are attributed to *ν* (NH_3_) and *ν*(antisymmetric NH_2_ def. of NH_4_^+^) at 0.1 V,^46^ and their strength gradually increases with decreasing potential. Additionally, the increase in these two bands' intensity in Cu–Cu_2_O/Ni(OH)_2_ is significantly higher than that in Cu–Cu_2_O catalysts. The above results show that NO_2_^−^ is quickly converted into NH_3_ on Cu–Cu_2_O/Ni(OH)_2_ catalyst, indicating its high catalytic activity for the electrocatalytic reduction of NO_3_^−^ to NH_3_.

We used 5,5-dimethyl-1-pyrrolineN-oxide (DMPO) as the *H capture agent and examined the capability of Ni(OH)_2_, Cu–Cu_2_O, and Cu–Cu_2_O/Ni(OH)_2_ to produce active hydrogen in the absence of NO_3_^−^*via* EPR. As shown in Fig. 7c, the H-DMPO peak intensity for Cu–Cu_2_O/Ni(OH)_2_ is significantly higher than that of Cu–Cu_2_O, but close to that of Ni (OH)_2_, indicating that the introduction of Ni(OH)_2_ improves the activation of the material for water. Subsequently, we probed whether the *H produced on the material was involved in the hydrogenation process of electrocatalytic nitrate reduction intermediates by monitoring the EPR spectrum of the electrolyte after introducing NO_3_^−^ (Fig. 7d). In the presence of NO_3_^−^, little change was observed in the H-DMPO signals for Ni(OH)_2_ and Cu–Cu_2_O, while the H-DMPO signal for Cu–Cu_2_O/Ni(OH)_2_ was significantly attenuated. This result indicates that the *H generated on the Cu–Cu_2_O/Ni(OH)_2_ surface during the catalytic hydrogenation process is rapidly consumed by the nitrogen-containing intermediate species. Nevertheless, Ni(OH)_2_ exhibits minimal activity in the electrocatalytic reduction of nitric acid, possibly attributed to its inadequate adsorption of nitric acid.

We utilized *in situ* Fourier transform infrared (FTIR) spectroscopy to investigate potential intermediates on the surfaces of Ni(OH)_2_, Cu–Cu_2_O, and Cu–Cu_2_O/Ni(OH)_2_ under different applied potentials. *Ex situ* infrared tests were conducted on standard samples (NO_3_^−^, NO_2_^−^, NH_4_^+^) to further monitor potential intermediates on these surfaces (Fig. S24–S26). The experimental findings revealed that in the presence of NO_3_^−^, distinct peaks corresponding to N–O stretching vibration of NO_3_^−^, N–H bending vibration of NH_4_^+^, and O–H bending vibration of H_2_O appeared at 1348 cm^−1^, 1452 cm^−1^, and 1647 cm^−1^Fig. 7e for Cu–Cu_2_O/Ni(OH)_2_ as the potential changed from 0.77 V to −0.47 V (*vs.*RHE).^47^ Meanwhile, only peaks corresponding to the bending vibrations of NO_3_^−^ and H_2_O were observed in the Ni(OH)_2_ system alone without detecting an NH_4_^+^ peak (Fig. S27, SI).^48^ In the Cu–Cu_2_O system (Fig. S28, SI), a peak at 1232 cm^−1^ attributed to N–O bending vibration of NO_2_^−^ was clearly observed but was absent in the Cu–Cu_2_O/Ni(OH)_2_ catalytic system. This indicates that NO_2_^−^ is further reduced and rapidly consumed in the Cu–Cu_2_O/Ni(OH)_2_ catalytic system, consistent with the results shown in Fig. 6a. Additionally, time-resolved *in situ* infrared spectroscopy FTIR of isotope labeling experiments also clearly observed the N–H bending vibration of NH_4_^+^ at 1452 cm^−1^ (Fig. S29, SI). Fig. 7f showed a broad peak at approximately 1647 cm^−1^, which is ascribed to *δ*H_2_O species in a solution without NO_3_^−^,^49^ indicating the occurrence of water splitting in the solution. The above results suggest that in the electrocatalytic reduction of NO_3_^−^ by Cu–Cu_2_O/Ni(OH)_2_, the Cu–Cu_2_O site predominantly catalyzes the generation of NO_2_^−^, while the incorporation of Ni(OH)_2_ site leads to a substantial production of *H, which synergistically enhances the formation of NH_3_, resulting in outstanding electrocatalytic performance of Cu–Cu_2_O/Ni(OH)_2_ in nitrate reduction.

### Theoretical validation of reaction pathways and synergistic effects

**Fig. 8:**
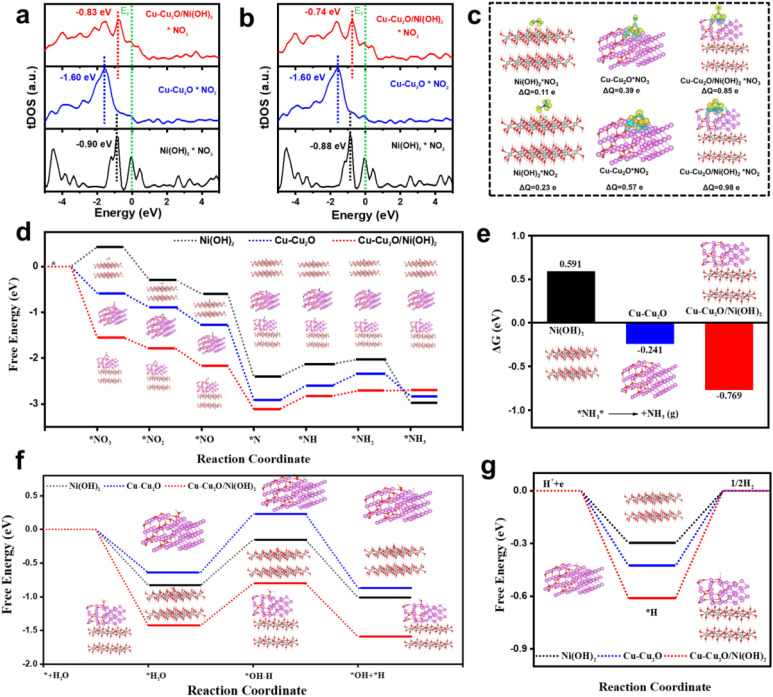
DFT calculations elucidating the reaction mechanism and synergy. Total density of states of Cu–Cu_2_O, Ni(OH)_2_, and Cu–Cu_2_O/Ni(OH)_2_ after adsorption of (a) *NO_3_ and (b) *NO_2_, respectively. *E*_F_ denotes the Fermi level. The short-dashed lines with distinct colors are the position of the d-band center, respectively. (c) The differential charge density of the adsorption structure of Cu–Cu_2_O, Ni(OH)_2_ and Cu–Cu_2_O/Ni(OH)_2_ for NO_3_^−^ and NO_2_^−^ and the corresponding electron transfer number. The value of the isosurface is 0.002 e/Å^3^. Yellow is the area where electrons accumulate and blue is the area where electrons dissipate. (d) Reaction free energies for different intermediates on a Cu–Cu_2_O, Ni(OH)_2_, and Cu–Cu_2_O/Ni(OH)_2_ catalyst surface. (e) The free energy change of NH_3_ desorption on Cu–Cu_2_O, Ni(OH)_2_, and Cu–Cu_2_O/Ni(OH)_2_. (f) Free energy change of reaction for the activation of Cu–Cu_2_O, Ni(OH)_2_, and Cu–Cu_2_O/Ni(OH)_2_ for the production of *H on water. (g) Free energy change for the production of H_2_ on Cu–Cu_2_O, Ni(OH)_2_ and Cu–Cu_2_O/Ni(OH)_2_.

Density functional theory (DFT) calculations were performed to gain atomic-level insight into the enhanced eNITRR activity. The reliability and convergence of the DFT calculations were validated by systematically examining the *U* value, *k*-point sampling, cutoff energy, slab model construction (fixed layers indicated by dashed lines), and the convergence of total energy with respect to vacuum layer thickness (Fig. S30–S33, SI). Models for Cu–Cu_2_O, Ni(OH)_2_, and the Cu–Cu_2_O/Ni(OH)_2_ heterojunction were constructed (Fig. S34 and Table S3, SI). The calculated energies of different species on various catalysts are presented in Tables S4–S7. The calculated d-band centers (Fig. 8a and b) for Cu–Cu_2_O/Ni(OH)_2_ shifted closer to the Fermi level compared to the individual components, indicating optimized adsorption strength for NO_3_^−^ and NO_2_^−^ intermediates. Differential charge density analysis (Fig. 8c) revealed that the charge transfer from Cu–Cu_2_O/Ni(OH)_2_ to NO_3_^−^ (0.85 e^−^) and NO_2_^−^ (0.98 e^−^) was substantially greater than that from Cu–Cu_2_O (0.39 e^−^, 0.57 e^−^) or Ni(OH)_2_ (0.11 e^−^, 0.23 e^−^), confirming the heterojunction's superior ability to activate these species.^50^

According to previous reports, the electrochemical reaction coordinates of NO_3_^−^ to NH_3_ involve deoxidation and hydrogenation processes. Fig. 8d, S35 and S37 (SI) illustrate a full eNITRR pathway in 0.1 M KOH (pH = 13) at 0 V *versus* Standard Hydrogen Electrode (SHE). For the adsorption of *NO_3_, Cu–Cu_2_O and Cu–Cu_2_O/Ni(OH)_2_ exhibit lower free energies (Δ*G* = −0.58 eV and Δ*G* = −1.55 eV), while Ni(OH)_2_ demonstrates the highest free energy change (0.43 eV). On the Cu–Cu_2_O surface, the potential-dependent reduction of *N to *NH step has the highest free energy change of 0.32 eV. In contrast to Cu–Cu_2_O, the introduction of Ni(OH)_2_ promotes the hydrogenation process (Δ*G* = +0.29 eV) of *N reduction to *NH. The findings indicate that Cu–Cu_2_O/Ni(OH)_2_ facilitates the adsorption of *NO_3_ and the reduction of *N to *NH step. Compared with Cu–Cu_2_O and Ni(OH)_2_, Cu–Cu_2_O/Ni(OH)_2_ has a lower activation energy in the overall reaction path, which is more conducive to catalyzing the overall process of NO_3_^−^ reduction to NH_3_. In addition, Cu–Cu_2_O/Ni(OH)_2_ is more conducive to the desorption of NH_3_, which is a thermodynamically favorable step (Fig. 8e).

For the eNITRR process, the activation of water to promote its gradual hydrogenation process is an important process to improve the performance of the catalyst. Therefore, we calculate the activation process of different catalysts to water (Fig. S37, SI). As shown in Fig. 8f and g, the Cu–Cu_2_O catalyst necessitates a greater input of free energy change to activate water and generate *H, whereas the introduction of Ni(OH)_2_, a catalyst that facilitates water activation, requires less free energy change to achieve the same effect. However, Ni(OH)_2_ tends to bind with *H, leading to H_2_ evolution and subsequent *H consumption, which hinders the progress of eNITRR. In contrast, the Cu–Cu_2_O/Ni(OH)_2_ catalyst exhibits lower free energy change for water activation to generate *H and demonstrates a higher free energy change in the subsequent combination of *H to form H_2_. This indicates that the Cu–Cu_2_O/Ni(OH)_2_ catalyst is more probable than the other catalysts to utilize the generated *H to promote the eNITRR hydrogenation reduction process.

## Conclusions

In summary, we have developed a widely applicable redox-potential-mediated strategy for the precise synthesis of 0D/2D Cu–Cu_2_O/MO_*x*_(OH)_*y*_ (M = Ni, Fe, Mn, Co, Cr) heterojunctions. By leveraging the inherent differences in standard electrode potentials between Cu and transition metals M, we achieved selective oxidation and phase separation, resulting in the *in situ* formation of well-defined heterointerfaces with atomic-level precision. The more oxidizable M species are preferentially oxidized and hydrolyzed into ultrathin MO_*x*_(OH)_*y*_ nanosheets, while Cu undergoes controlled oxidation to form Cu–Cu_2_O nanoparticles epitaxially anchored on the nanosheets. As a proof of concept, the Cu–Cu_2_O/Ni(OH)_2_ heterojunction exhibits exceptional electrocatalytic performance for the nitrate reduction reaction (eNITRR), achieving a Faradaic efficiency of 98.15% and an outstanding ammonia yield rate of 12,974.5 µg cm^−2^ h^−1^ at a low catalyst loading of 1 mg cm^−2^, surpassing most reported catalysts. Integrated experimental and theoretical analyses reveal that the superior performance originates from synergistic interfacial effects: Cu–Cu_2_O facilitates nitrate adsorption and activation, while Ni(OH)_2_ efficiently cleaves H_2_O to generate active *H species for the rapid hydrogenation of nitrogen intermediates. The catalyst also demonstrates remarkable stability over 140 hours of continuous operation. This work provides a generalizable synthesis strategy and deepens the understanding of interface engineering at the atomic scale, offering a powerful platform for designing advanced electrocatalysts for sustainable chemical synthesis and energy conversion.

## Experimental

### Materials

Copper(ii) chloride dihydrate (CuCl_2_·2H_2_O, AR), nickel chloride hexahydrate (NiCl_2_·6H_2_O, AR), sodium nitrate (NaNO_3_, AR), and anhydrous sodium sulfate (Na_2_SO_4_, AR) were obtained from Chron Chemicals LTD. Ethanol (AR), *n*-hexane (AR), and acetone (AR) were supplied by Lianlong Bohua (Tianjin) Pharmaceutical Chemistry Co., LTD. Sodium oleate (98.0%), isotopically labeled sodium nitrate-^15^ N, and Nafion aqueous solution (5.0 wt%) were purchased from Macklin. Carbon felt, used as the substrate, was acquired from Sinero Technology Co., LTD. Prior to use, the carbon felt was cleaned *via* sequential ultrasonic washing with isopropanol, ethanol, and deionized water.

#### Synthesis of Cu nanoparticles

The complex precursor of Cu was prepared by hydrothermal method, and then the Cu nanoparticles were reduced in organic solvent under the action of ligands. 0.171 g (0.1 M) CuCl_2_·2H_2_O was firstly added to 30 mL deionized water, then continually added 40 mL ethanol and 70 mL *n*-hexane. Then 1.218 g (0.4 M) sodium oleate was also added into the above solution, stirred and refluxed at 70 °C for 8 h. After the solution was cooled, the solution was divided into two layers. The precursor of Cu complex was obtained by washing the upper solution in the divided solution. 10 mL of the precursor solution of Cu complex was measured and added to the mixed solution of 50 mL ethanol and 50 mL *n*-hexane, and then the Cu nanoparticles were rapidly reduced under the reduction of NaBH_4_ (50 mg). Finally, the solution was centrifuged, washed and dried to obtain the Cu nanoparticles.

#### Synthesis of Ni nanoparticles

The synthesis method of nickel nanoparticles is similar to the above method. 0.238 g (0.1 M) NiCl_2_·6H_2_O was firstly added to 30 mL deionized water, then continually added 40 mL ethanol and 70 mL *n*-hexane. Then 1.218 g (0.4 M) sodium oleate was also added into the above solution, stirred and refluxed at 70 °C for 8 h. After the solution was cooled, the solution was divided into two layers. The precursor of Ni complex was obtained by washing the upper solution in the divided solution. 10 mL of the precursor solution of Ni complex was measured and added to the mixed solution of 50 mL ethanol and 50 mL *n*-hexane, and then the Ni nanoparticles were rapidly reduced under the reduction of NaBH_4_ (50 mg). Finally, the solution was centrifuged, washed and dried to obtain the Ni nanoparticles.

#### Synthesis of CuNi nanoparticles

Take the precursor solution prepared above, 5 mL of the precursor solution of Ni complex and 5 mL of the precursor solution of Cu complex were measured and added to the mixed solution of 50 mL ethanol and 50 mL *n*-hexane, and then the CuNi nanoparticles were rapidly reduced under the reduction of NaBH_4_ (50 mg). Finally, the solution was centrifuged, washed and dried to obtain the CuNi nanoparticles.

#### Synthesis of Cu/Cu_2_O nanoparticles

The prepared Cu nanoparticles were weighed 10 mg and added to 10 mL deionized water, and ultrasounded at 35 °C for 4 h. After ultrasound, the surface of Cu nanoparticles was partially oxidized to form Cu–Cu_2_O nanoparticles. Finally, the solution after ultrasound is centrifuged, washed and dried to obtain Cu–Cu_2_O nanoparticles.

#### Synthesis of Ni(OH)_2_ nanosheets

The prepared Ni nanoparticles were weighed 10 mg and added to 10 mL deionized water and ultrasounded at 35 °C for 4 h. Due to hydrolysis, Ni nanoparticles were gradually hydrolyzed into Ni(OH)_2_ nanosheets. Finally, the ultrasonic solution was centrifuged, washed and dried to obtain Ni(OH)_2_ nanosheets.

#### Synthesis of Cu–Cu_2_O/Ni(OH)_2_ nanosheets

The prepared CuNi nanoparticles were weighed at 10 mg and added to 10 mL of deionized water and ultrasounded at 35 °C for 4 h. Due to hydrolysis, CuNi nanoparticles formed Ni(OH)_2_ nanosheets *in situ* and loaded Cu–Cu_2_O nanoparticles at the same time. Finally, the ultrasonic solution was centrifuged, washed and dried to obtain Cu–Cu_2_O/Ni(OH)_2_ nanosheets.

#### Electrocatalytic performance measurements

The electrocatalytic activity measurements were performed in an open two-compartment H-cell separated. Electrode preparation: 1 milligram of the catalyst were ultrasonically dispersed in a mixed solution 0.95 mL of isopropyl alcohol and 50 µL of Nafion solution (5 wt%). All of the dispersed catalyst slurry was dropped onto 1 cm^2^ of carbon felt (Except for the specified conditions) as the work electrode, followed by drying at 60 °C for 4 h. A Platinum tablet (1*1 cm^2^) and a SCE (filled with saturated KCl) were used as counter and reference electrodes, respectively. The 30 mL NaOH (0.1 M) solution was used as the anodic electrolyte and the 30 mL NaOH (0.1 M)/NaNO_3_–N (500 mg L^−1^) solution was used as the cathodic electrolyte. The electrolyte in the cathodic compartment was stirred at a rate of 200 rpm during electrolysis in a 28 °C water bath. All of the electrocatalytic nitrate reduction reaction (eNITRR) experiments were performed using a three-electrode system connected to an electrochemical workstation (CHI 760E). The pH is 13.0 in this mixing electrolyte system. All potentials measured were calibrated to *vs.* RHE (reversible hydrogen electrode) using the following formula.*E* (RHE) = *E* (SHE) + 0.0592 × pH + 0.24

#### ECSA measurements

The cycle voltammetry profiles were obtained using a glassy carbon electrode (GCE, 0.071 cm^2^) deposited 10 µL catalyst ink as the working electrode. The catalyst ink formula: 10 mg of the catalyst was ultrasonically dispersed in a mixed solution 0.95 mL of isopropyl alcohol and 50 µL of Nafion solution (5 wt%).

#### ^15^NO_3_^−^ isotope labeling experiments quantification by 1H NMR

^1^H NMR spectroscopy (400 MHz) was used to detect ammonia in the isotope-labelling measurement. The generated NH_3_ was collected in the HCl solution. Next, all of the sample solutions were rotary evaporated to remove water and excess HCl, a white powder sample was obtained. Then, the power sample was mixed with 5 mg maleic acid (C_4_H_4_O_4_, internal standard) and dissolved in 0.5 mL DMSO-d6 (hexadeuterodimethyl sulfoxide). Finally, the prepared mixture was tested by a JNM-ECS 400 M spectrometer at ambient conditions and the NH_3_ product peaks were analyzed.

## Author contributions

Tuo Zhang: data curation, formal analysis, investigation methodology, software, writing – original draft; Tianzhi Hao: data curation, formal analysis, investigation, methodology, writing – original draft; Xiangyang Hou: software, writing – original draft, Yuhui Yin: data curation, investigation, methodology, software, writing – original draft; Guowen Hu: data curation, investigation, methodology, writing – original draft; Genping Meng: data curation, investigation, methodology, software, writing – original draft; Shihao Sun: data curation, investigation, methodology, software, writing – original draft; Hua Li: data curation, investigation, methodology, software, writing – original draft; Baodui Wang: conceptualization, formal analysis, investigation, methodology, resources, funding acquisition, supervision, project administration, and writing – review & editing.

## Conflicts of interest

There are no conflicts to declare.

## Supplementary Material

SC-OLF-D5SC08998K-s001

## Data Availability

All the data supporting this article have been included in the main text and the supplementary information (SI). Supplementary information is available. See DOI: https://doi.org/10.1039/d5sc08998k.
