# Supplementary material for: Atomic-scale redox-potential-mediated engineering of 0D/2D Cu–Cu2O/MOx(OH)y heterojunctions for efficient nitrate electroreduction to ammonia
Source: Chem Sci. 2026 Jan 23;17(11):5661–72. doi: 10.1039/d5sc08998k (PMC12835886; doi:10.1039/d5sc08998k)
Supplement: SC-017-D5SC08998K-s001 [file SC-017-D5SC08998K-s001.pdf]

Supplementary Information (SI) for Chemical Science.
This journal is © The Royal Society of Chemistry 2026

Supporting Information

Atomic-scale redox-potential-mediated engineering of 0D/2D Cu–$Cu_2O/MO_x(OH)_y$ heterojunctions for efficient nitrate electroreduction to ammonia

Tuo Zhang,[‡a] Tianzhi Hao,[‡a] Xiangyang Hou,[b] Yuhui Yin,[a] Guowen Hu,[a] Genping Meng,[a] Shihao Sun, [a] Hua Li*[a] and Baodui Wang*[a]

a. State Key Laboratory of Natural Product Chemistry, College of Chemistry and Chemical Engineering, Lanzhou University, Lanzhou, Gansu 730000, China.

b. Department of Chemistry and Biochemistry, Florida International University.

E-mail: huali@lzu.edu.cn; wangbd@lzu.edu.cn

This file includes:

Experimental Section
Compound Characterization
Supplementary Tables
Fig.S1 to S31
References (1 to 14)

Experimental Section

Instruments

The morphologies of the samples were analyzed transmission electron microscope (TEM, FEI, Talos 200s). The structures of the samples were characterized through X-ray powder diffraction (XRD) patterns (Bruker AXS D8-Advance diffractometer with Cu Kα radiation). X-ray photoelectron spectroscopy (XPS) was performed on a PHI-5702 multifunctional spectrometer with Al Kα radiation. All LSV measurements were performed with 80% iR-compensation applied during the testing via the positive feedback method, where the solution resistance (R_s) was determined by electrochemical impedance spectroscopy (EIS) at the open-circuit potential. Ion chromatography (IC, Qingdao ELEN Technology YC7000) was used to detect the ionic products, such as NH_4^+ and NO_3^- /NO_2^-. ^{1}H NMR was carried out with Liquid Nuclear Magnetic Resonance Spectrometer (JNM-ECS 400M). All electrochemical measurements were performed by an electrochemical workstation (CHI 760E) in an H-type cell separated by a treated Nafion 117 membrane. In situ Raman spectra were conducted on a Raman spectrometer (LabRAM Soleil) using an excitation of 532 nm laser; the potential was controlled by an electrochemical workstation (CHI 660E).

Determination of products

Detection of nitrate-N and nitrite-N by ion chromatography (IC)

Before each electrochemical test, the electrolyte was bubbled with high-purity Ar for 30 min to ensure an anaerobic environment at a flow rate of 20 sccm, and an Ar atmosphere was maintained during the entire experimental process. All constant potential electrolysis tests were conducted in an H-type cell with 30 mL of electrolyte in each chamber. To ensure efficient mass transport, the catholyte was continuously stirred at 200 rpm using a magnetic stirrer. For ion chromatography (IC) analysis, the injection volume was set at 25 μL. All UV-vis spectrophotometric measurements were performed using a 1 cm path-length quartz cuvett

In order to adjust the absorbance to match the range of calibration curves, the electrolytes were diluted 100 times. Then NO_3^- and NO_2^- were detected using IC. NO_3^- peaks were at 17.8 minutes and NO_2^- peaks were at 12.1 minutes. The NO_3^- and NO_2^- concentration were obtained by bringing the peak area into the standard curve. The standard curves were determined by preparing different concentrations of the standard NO_3^--N (1000 mg L^{-1}) and NO_2^-N (1000 mg L^{-1}).

Detection of ammonia-N by ion chromatography (IC)

In order to adjust the absorbance to match the range of calibration curves, the electrolytes were diluted 100 times. Then NH_4^+ was detected using ion chromatography (IC). NH_4^+ peaks were at 6.7 minutes. The NH_4^+ concentration was obtained by bringing the peak area into the standard curve. The standard curves were determined by preparing different concentrations of the standard NH_4^+-N (1000 mg L^{-1}).

Determination of products by coloration method

(1) Detection of Ammonium Ion (NH_4^+) Concentration. The concentration of ammonium ions (NH_4^+) in the solution was determined using a spectrophotometric method. This involved a

colorimetric reaction with NH_4^+ ions, followed by quantitative analysis using a UV-visible spectrophotometer to construct an absorbance-concentration calibration curve.

(1.1) Preparation of Reagents

Phenol-Ethanol Solution: Dissolve 62.5 g of refined, colorless phenol in 45 mL of ethanol. Store the solution in a refrigerator.

Sodium Nitroprusside Solution (10 g/L): Dissolve 1 g of sodium nitroprusside ($Na_2[Fe(CN)_5NO]\cdot H_2O$) in deionized water, and dilute to a final volume of 100 mL. Store in a refrigerator.

Sodium Hydroxide Solution (240 g/L): Dissolve 120 g of sodium hydroxide in deionized water and dilute to a final volume of 500 mL.

Sodium Citrate Solution (400 g/L): Dissolve 200 g of sodium citrate ($C_6H_5Na_3O_7$) in deionized water and dilute to a final volume of 500 mL.

Phenol-Salt-Citrate Solution: Mix 3.0 mL of sodium nitroprusside solution, 5.0 mL of phenol-ethanol solution, 6.5 mL of sodium hydroxide solution, and 50 mL of sodium citrate solution thoroughly. Store in a refrigerator.

Chlorinated Buffer Solution: Dissolve 12 g of anhydrous sodium carbonate (Na_2CO_3) and 0.8 g of sodium bicarbonate ($NaHCO_3$) in 100 mL of deionized water. Add 34 mL of sodium hypochlorite solution (30 g/L) and dilute to a final volume of 200 mL.

(1.2) Preparation of Standard Solutions

Ammonia Nitrogen Standard Stock Solution (NH_3-N concentration of 1.00 mg/mL): Dry 3.8190 g of ammonium chloride (NH_4Cl) in an oven at 105°C for 1 hour, dissolve in deionized water, and dilute to a final volume of 1000 mL.

Ammonia Nitrogen Standard Working Solution (NH_3-N concentration of 5 μg/mL): Transfer 5.00 mL of the ammonia nitrogen standard stock solution to a 1000 mL volumetric flask and dilute to the mark with deionized water.

Preparation of Standard Series: Transfer 0.00 mL, 0.05 mL, 0.10 mL, 0.50 mL, 1.00 mL, 1.50 mL, 2.00 mL, and 4.00 mL of the ammonia nitrogen standard working solution to eight different colorimetric tubes and dilute with deionized water to a final volume of 10 mL in each tube.

(1.3) Construction of Standard Curve

Add 1.0 mL of phenol-salt-citrate solution to each standard tube, followed immediately by 0.4 mL of chlorinated buffer solution. Mix thoroughly and allow to stand for 90 minutes. Using a UV-visible spectrophotometer with a 1 cm pathlength and deionized water as a reference, scan the absorbance spectra of the solutions from 900 to 500 nm to determine the UV absorbance at 630 nm.

For the determination of NH_4^+ concentration in post-electrochemical reaction solutions, minimize interference with the electrochemical system by using the smallest possible volume of electrolyte solution for measurement. Transfer 0.1 mL of the post-reaction electrolyte solution, dilute it with deionized water to a final volume of 1 mL, and then transfer 0.1 mL of this diluted solution to another dilution with deionized water to a final volume of 2.5 mL. Add 0.25 mL of phenol-salt-citrate solution and 0.1 mL of chlorinated buffer solution. Mix thoroughly, let stand for 90 min, and

measure the absorbance at 630 nm using the method described above. Calculate the concentration based on the standard curve.

(2) Detection of Nitrite Ion (NO_2^-) Concentration

The concentration of nitrite ions (NO_2^-) in the solution was determined using the diazo-coupling spectrophotometric method. This method involves the diazotization of NO_2^- in water with p-aminobenzenesulfonamide and subsequent coupling with N-(1-naphthyl)ethylenediamine (NEDD) to produce a reddish-violet azo dye, which is quantified by colorimetric analysis.

(2.1) Preparation of Reagents

p-Aminobenzenesulfonamide Solution (10 g/L): Dissolve 5 g of p-aminobenzenesulfonamide in 350 mL of hydrochloric acid solution (volume ratio: water: hydrochloric acid = 6:1) and dilute to a final volume of 500 mL with deionized water.

NEDD Solution (1 g/L): Dissolve 0.2 g of NEDD in 200 mL of pure water and store it in a refrigerator.

(2.2) Preparation of Standard Solutions

Nitrite Nitrogen Standard Stock Solution (NO_2^--N concentration of 50 μg/mL): Dry 0.2463 g of sodium nitrite ($NaNO_2$) in a desiccator for 24 hours. Dissolve in deionized water and make up to a final volume of 1000 mL, adding 2 mL of chloroform to preserve stability.

Nitrite Nitrogen Standard Working Solution (NO_2^--N concentration of 0.10 μg/mL): Transfer 1.00 mL of the nitrite nitrogen standard stock solution to a 500 mL volumetric flask and dilute to the mark with deionized water.

Preparation of Standard Series: Transfer 0 mL, 0.25 mL, 0.50 mL, 1.25 mL, 2.50 mL, 3.75 mL, 5.00 mL, and 6.25 mL of the nitrite nitrogen standard working solution into 25 mL colorimetric tubes and dilute to 25 mL with deionized water.

(2.3) Construction of Standard Curve

Add 0.5 mL of p-aminobenzenesulfonamide solution to each standard tube, mix, and let stand for 2 to 8 minutes. Then, add 0.5 mL of NEDD, mix thoroughly, and measure the absorbance using a UV-visible spectrophotometer with a 1 cm pathlength, using deionized water as a reference. Scan the absorbance spectra from 800 to 400 nm to determine the absorbance at 540 nm.

For the determination of NO_2^- concentration in post-electrochemical reaction solutions, transfer 0.1 mL of the reaction electrolyte solution and dilute it with deionized water to a final volume of 1 mL. From this diluted solution, transfer 0.1 mL to another dilution with deionized water to a final volume of 5.0 mL. Add 0.1 mL of p-aminobenzenesulfonamide solution, mix, and let stand for 2 to 8 minutes. Then, add 0.1 mL of NEDD, mix thoroughly, and measure the absorbance at 540 nm using the method described above. Calculate the concentration based on the standard curve.

(3) Detection of Nitrate Ion (NO_3^-) Concentration

The concentration of nitrate ions (NO_3^-) in the solution was determined using the UV spectrophotometric method. This method takes advantage of the property that NO_3^- absorbs UV light at 220 nm but not at 275 nm.

(3.1) Preparation of Standard Solutions

Nitrate Nitrogen Standard Stock Solution (NO_3^--N concentration of 100 μg/mL): Dry 0.7218 g of potassium nitrate (KNO_3) at 105°C for 2 hours. Dissolve in deionized water and make up to a final volume of 1000 mL, adding 2 mL of chloroform to maintain stability.

Nitrate Nitrogen Standard Working Solution (NO_3^--N concentration of 10 μg/mL): Transfer 10 mL of the nitrate nitrogen standard stock solution to a 100 mL volumetric flask and dilute to the mark with deionized water.

Preparation of Standard Series: Transfer 0 mL, 0.50 mL, 2.50 mL, 5.00 mL, 10.00 mL, 15.00 mL, and 17.50 mL of the nitrate nitrogen standard working solution into 25 mL colorimetric tubes. Dilute to 25 mL with deionized water and add 0.5 mL of hydrochloric acid (volume ratio: hydrochloric acid: water = 1:11) to prepare a nitrate-nitrogen standard series ranging from 0 to 7 mg/L.

(3.2) Construction of Standard Curve

Using a UV-visible spectrophotometer with a 1 cm path length and deionized water as a reference, scan the absorbance spectra of the standard series from 300 to 190 nm. Specifically, measure the absorbance at 220 nm and 275 nm to determine the NO_3^- concentration.

For the determination of NO_3^- concentration in post-electrochemical reaction solutions, transfer 0.1 mL of the reaction electrolyte solution to a dilution with deionized water to a final volume of 1 mL. From this diluted solution, transfer 0.1 mL to another dilution with deionized water to a final volume of 5.0 mL. Add 0.1 mL of hydrochloric acid (volume ratio: hydrochloric acid: water = 1:11), mix thoroughly, and measure the absorbance at 220 nm and 275 nm using the method described above. Calculate the NO_3^- concentration based on the standard curve.

In-situ Raman measurements

The in-situ Raman measurements were performed on Raman Spectrometer (LabRAM Soleil) using an excitation of 532 nm laser, the potential was controlled by an electrochemical workstation (CHI 660E). The catalyst ink was dropped on a glassy carbon electrode as a working electrode to keep the plane of the sample perpendicular to the incident laser. A platinum wire and an Ag/AgCl electrode (filled with saturated KCl) were used as counter and reference electrodes, respectively. A mixed solution (0.1 M NaOH + 0.5 M $NaNO_3$) was used as the electrolyte.

Calculation of yield, selectivity, and Faradaic efficiency

All results were calculated from peak areas measured by IC and coloration method.

The result of yield is obtained by Eq. 1:

$$Yiled_{NH_3} = \left(c_{NH_3} \times V\right)/(t \times m) \#(1)$$

The result of conversion rate is obtained by Eq. 2:

$$Conversion_{NO_3^-} = \Delta c_{NO_3^-} / c_0 \#(2)$$

The result of NO_2^- selectivity is obtained by Eq. 3:

$$Selectivity = c_{NO_2^-} / \Delta c_{NO_3^-} \#(3)$$

The result of NH_3 selectivity is obtained by Eq. 4:

$Selectivity = c_{NH_3} / \Delta c_{NO_3^-}$ #(4)

The Faradaic efficiency (FE) of nitrate reduction was obtained by Eq 5:

$FE = (8F \times c_{NH_3} \times V)/(M_{NH_3} \times Q)$#(5)

Here, c_{NH_3} is the mass concentration of NH_3 in the cathode electrolyte after the test, V is the volume of electrolyte in the cathode, M_{NH_3} is the molar mass of NH_3, t is the electrocatalytic time, m is the mass of the catalyst on the working electrode, Δc is the difference in the NO_3^- concentration of nitrate in the catholyte before and after electrocatalytic, c_0 is the actual concentration of nitrate in the electrolyte, c is the concentration of nitrite or ammonia in the catholyte after electrolysis, F is the Faradaic constant (96485 C mol^{-1}), Q is the total charge pass electrode during the electrochemical process.

Density functional theory calculations

We have employed the first-principles to perform all density functional theory (DFT) calculations within the generalized gradient approximation (GGA) using the Perdew-Burke-Ernzerhof (PBE) formulation. [1,2,3] We have chosen the projected augmented wave (PAW) potentials to describe the ionic cores and take valence electrons into account using a plane wave basis set with a kinetic energy cutoff of 450 eV. [4,5] Partial occupancies of the Kohn−Sham orbitals were allowed using the Gaussian smearing method and a width of 0.05 eV. The electronic energy was considered self-consistent when the energy change was smaller than 10^{-6} eV. A geometry optimization was considered convergent when the force change was smaller than 0.05 eV $Å^{-1}$. All calculations were performed within a spin-polarized framework to ensure an accurate description of the electronic states, with ISPIN set to 2 and default initial magnetic moments applied. The vacuum spacing along the direction perpendicular to the plane of the structure exceeds 15 Å. The U correction for Cu and Ni atoms had been applied with 4eV and 5eV. Given the strict convergence of adsorption energies and their established theoretical relationship with key electronic descriptors (e.g., d-band center), the reported electronic-structure properties are reliable under the selected parameters. The Brillouin zone integration is performed using 2×2×1 Monkhorst-Pack k-point sampling for a structure. Finally, the adsorption energies (Eads) were calculated as Eads= Ead/sub -Ead -Esub, where Ead/sub, Ead, and Esub are the total energies of the optimized adsorbate/substrate system, the adsorbate in the structure, and the clean substrate, respectively. The Gibbs free energy (G) of all intermediates was obtained by applying thermodynamic corrections to the DFT electronic energy (E_{DFT}) according to:

$$G = E_{DFT} + ZPE - TS \quad (T = 298.15\ K)$$

where E_{DFT} is the difference of electron energies calculated by DFT; ZPE and S are the changes of zero-point energy and entropy, respectively, which are obtained from vibrational frequencies. T is

the temperature (298.15 K). The reported free energy changes correspond to standard conditions (pH = 0). Although the actual reaction environment is pH-dependent, with the correction given by $\Delta G(pH) = \Delta G(pH=0) + kT \ln(10) \times pH$, explicit pH corrections were not applied at this stage to simplify the analysis and highlight intrinsic catalytic trends. This treatment effectively assumes identical pH dependences for all proton-involved steps, such that the resulting activity trends (e.g., overpotential ranking) remain qualitatively valid.

For the CHE approach, we explicitly adopted the computational hydrogen electrode model. The core of this model is to relate the free energy of a proton–electron pair ($H^+ + e^-$) in electrochemical steps to the standard hydrogen electrode. In our calculations, the standard free energy is defined as:

$$G(H^+ + e^-) = 1/2\ G(H_2)$$

This relation serves as the reference for evaluating the free energy changes (ΔG) of all reaction steps involving ($H^+ + e^-$) transfer. All reaction free energy diagrams presented in this work are constructed based on this model.

Gaseous HNO_2 was chosen as a reference to avoid calculating the energy of the charged NO_2^-.[6]

$$HNO_2(g) \rightarrow H^+ + NO_2^-$$

For NO_3^-, we applied exactly the same logic in our calculations.

This study aims to provide a systematic thermodynamic evaluation of the nitrate reduction reaction using DFT calculations. All discussions regarding the relative difficulty of reaction steps are based on the Gibbs free energy changes (ΔG) between adjacent stable intermediates. No transition-state searches were performed, and kinetic activation barriers were not calculated. The conclusions of this work are therefore drawn from thermodynamic analysis, while future studies involving kinetic barrier calculations are required for a more complete mechanistic understanding.

The charge transfer was quantified using the Bader partitioning scheme. While absolute atomic charges may vary depending on the partitioning algorithm used, the relative electronic trends and charge-flow directions discussed in this work are insensitive to the specific choice of the scheme, ensuring the robustness of our mechanistic insights.

The NO_3^- reduction reaction on designed catalysts surfaces were simulated according to the following reactions:

$$* + NO_3^- \rightarrow *NO_3 + e^-$$

$$*NO_3 + 2H^+ + 2e^- \rightarrow *NO_2 + H_2O$$

$$*NO_2 + 2H^+ + 2e^- \rightarrow *NO + H_2O$$

$$*NO + 2H^+ + 2e^- \rightarrow *N + H_2O$$

$$*N + H^+ + e^- \rightarrow *NH$$

$$*NH + H^+ + e^- \rightarrow *NH_2$$

$$*NH_2 + H^+ + e^- \rightarrow *NH_3$$

$$*NH_3 \rightarrow * + NH_3$$

where * represents the active site.

Theoretical modeling and simulation

The equilibrium lattice constants of Cu_2O, Cu, $Ni(OH)_2$ unit cell were optimized. We then use it to construct a Cu(111), $Cu_2O(111)$, $Ni(OH)_2(002)$ surface model. $Ni(OH)_2(002)$ surface structures

with two layers had been used. In addition, the Cu_2O-Cu structures were established using the Cu surface and Cu_2O surface with the lattice parameters (a=7.6383Å, b=13.7627Å, c=23.9756Å). The Cu_2O-Cu structures with 4-layer of Cu-O-Cu layers include 56 atoms. Cu-Cu_2O-$Ni(OH)_2$ structures had been established Cu_2O-Cu structures and $Ni(OH)_2$ surface with the lattice parameters (a=7.0121Å, b=13.2673Å, c=28.0317Å). During structural optimizations, a 2×2×1 k-point grid in the Brillouin zone was used for k-point sampling, and the bottom two atomic layers were fixed while other layers were allowed to relax to form stable structures.

Due to the activity of metal sites, our work also chooses metal sites as adsorption sites. For the structure of $Ni(OH)_2$, Ni metal sites are selected, and for the Cu- Cu_2O structure, Cu sites at the interface are selected for calculation to reflect the interface characteristics.

Supplementary Tables

Table S1. Element content determined by ICP-OES (mg/L)

Element	Before $_{\text{Mean value}}$	After $_{\text{Mean value}}$
Ni	5.039	4.983
Cu	1.007	1.006
Ratio (Cu:Ni)	1:5	1:4.95

The elemental composition of copper and nickel was analyzed before and after ultrasonication by inductively coupled plasma-optical emission spectroscopy (ICP-OES). As shown in Table S1, the composition ratio of CuNi NPs before ultrasound treatment is 1:5.00 for copper and nickel. The composition ratio of Cu-Cu_2O/$Ni(OH)_2$ formed after ultrasonic treatment remains at 1:4.95, indicating that the relative content between copper and nickel remained stable and did not undergo significant changes during the ultrasonic treatment process.

Table S2 Comparison of the performance of the present work with recently reported catalysts

Catalyst	Potential (V vs. RHE)	Yield of NH_3 (μg h^{-1} mg^{-1})	FE (%)	References
	-0.49	12974.5	85.3	
$Cu\text{-}Cu_2O/Ni(OH)_2$	-0.29	12087.4	91.3	This work
	0.01	3251.25	98.15	
$CoMn_2O_4$/NC	-0.7	1040.1	92.4	(7)
CuCl_BEF	-1	1820	88	(8)
Pd/TiO_2	-0.7	1120	92.1	(9)
Co-Fe@Fe_2O_3	-0.75	1505	85.2	(10)
Cu/Pd/CuO_x	-0.3	1513	84	(11)
PA-RhCu cNCs	0.05	2397	93.7	(12)
RuCu/Cu_2O@Ti_3C_2	-0.7	1792	48.3	(13)
CuPd/CN	-0.46	1536.8	96.13	(14)
Co_3O_4@CNF	-0.9	2340	92.7	(9)
BiN_2C_2	-0.35	1380	88.7	(10)
Cu–PTCDA	-0.4	440	77	(15)
Fe SAC	-0.66	2098	75	(16)
CoP/CC	-0.4	317	65	(17)
Fe MOF	-1	4250	90	(18)

Table S3. Calculated lattice parameters a, b, and c(Å), and the formation enthalpy of per atom ΔH (KJ/mol) for $Ni(OH)_2$, Cu-Cu_2O, Cu-$Cu_2O/Ni(OH)_2$, respectively

**Sample**	**a(Å)**	**b(Å)**	**c(Å)**	**Δ*H*(KJ/mol)**
$Ni(OH)_2$	12.772	12.772	19.992	-5.446
Cu-Cu_2O	7.638	13.763	23.976	-7.316
Cu-$Cu_2O/Ni(OH)_2$	7.012	13.267	28.032	-8.198

Table S4 Gibbs free energies of different species (eV).

NO_2	NO_3	O_2	H_2	HNO_3	H_2O
**17.858**	**25.348**	**-9.960**	**-6.800**	**-28.608**	**-14.220**

Table S5 Energies of different species under Cu-Cu_2O/Ni$(OH)_2$ (eV).

	E_{DFT}	ZPE	TS	G	$G_{correct}$	ΔG
Cu-Cu_2O/Ni$(OH)_2$	-481.496	0.000	0.000	-481.496	-506.844	
$*NO_3$	-508.563	0.725	0.553	-508.391	-508.391	-1.788
$*NO_2$	-503.716	0.553	0.489	-503.652	-508.632	-1.547
*NO	-499.353	0.489	0.193	-499.057	-509.017	-2.173
*N	-495.086	0.631	0.556	-495.011	-509.951	-3.107
*NH	-498.308	0.552	0.369	-498.125	-509.665	-2.821
$*NH_2$	-501.914	0.902	0.401	-501.413	-509.553	-2.709
$*NH_3$	-505.094	0.881	0.588	-504.801	-509.541	-2.697

Table S6 Energies of different species under Cu-Cu_2O (eV).

	E_{DFT}	ZPE	TS	G	$G_{correct}$	ΔG
Cu-Cu_2O	-364.910	0.000	0.000	-364.910	-390.258	
*NO_3	-390.924	0.559	0.472	-390.837	-390.837	-0.579
*NO_2	-386.255	0.482	0.396	-386.169	-391.149	-0.891
*NO	-381.905	0.553	0.221	-381.573	-391.533	-1.275
*N	-378.410	0.597	0.415	-378.228	-393.168	-2.910
*NH	-381.589	0.493	0.216	-381.312	-392.852	-2.594
*NH_2	-384.755	0.892	0.588	-384.451	-392.591	-2.333
*NH_3	-388.133	0.759	0.973	-388.347	-393.087	-2.829

Table S7 Energies of different species under $Ni(OH)_2$ (eV).

	E_{DFT}	ZPE	TS	G	$G_{correct}$	ΔG
$Ni(OH)_2$	-785.485	0.000	0.000	785.485	-810.833	
$*NO_3$	-810.397	0.260	0.270	810.407	-810.407	0.426
$*NO_2$	-806.397	0.450	0.200	806.147	-811.127	-0.294
*NO	-801.799	0.670	0.340	801.469	-811.429	-0.596
*N	-798.594	0.521	0.221	798.294	-813.234	-2.401
*NH	-801.638	0.330	0.110	801.418	-812.958	-2.125
$*NH_2$	-805.269	0.660	0.110	804.719	-812.859	-2.026
$*NH_3$	-808.925	0.690	0.830	809.065	-813.805	-2.972

Supplementary Figures

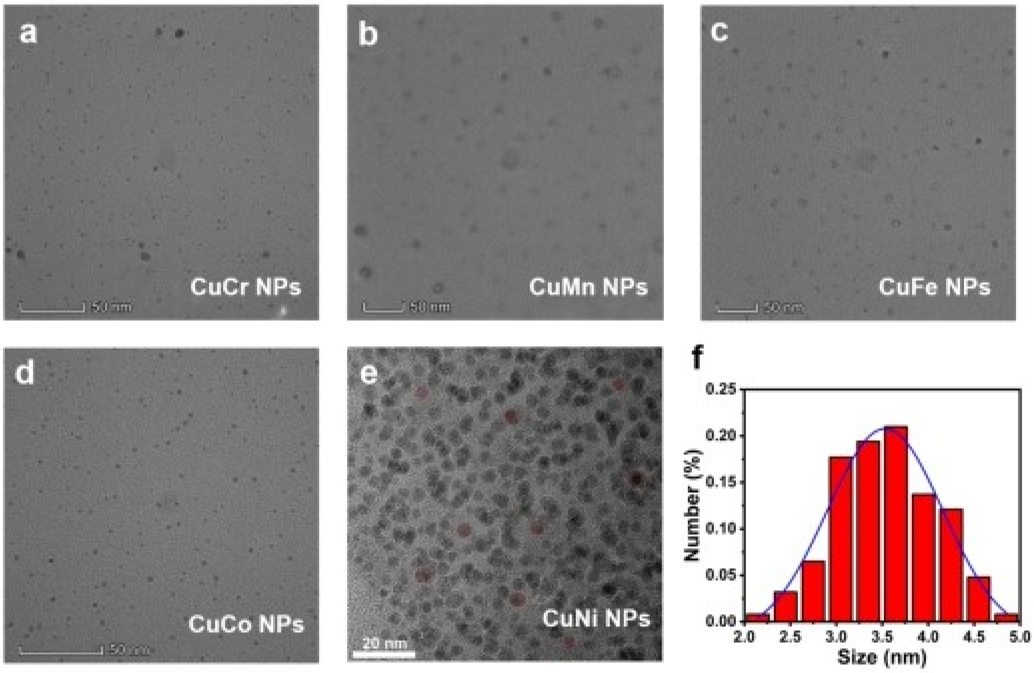

Fig. S1. Low-magnification TEM image of (a) CuCr nanoparticles, (b) CuMn nanoparticles, (c) CuFe nanoparticles, (d) CuCo nanoparticles, (e) CuNi nanoparticles, and (f) Statistical distribution of CuNi nanoparticles. The synthesized CuM (M = Cr, Mn, Fe, Co, Ni) nanoparticles are spherical in shape as shown in Figure S1, and the red circles with different diameters are shown as different sizes. The histogram of the particle size distribution shows that the diameter of the nanoparticles is about 3.89 nm (Figure S1, f).

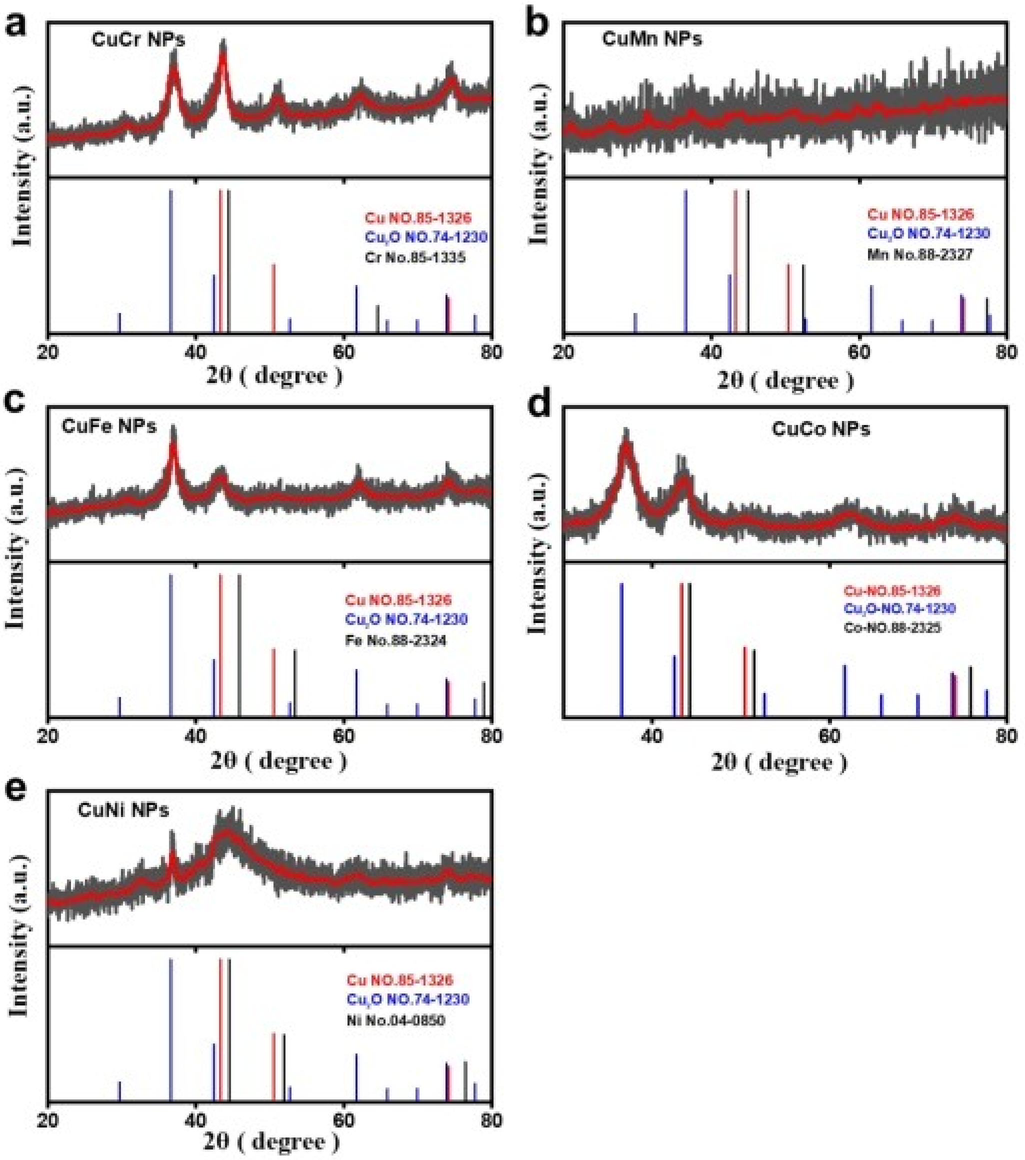

Fig. S2. XRD patterns of CuM nanoparticles. As shown in Figure S2, the X-ray powder diffraction (XRD) results are consistent with the established reference cards for CuM NPs. The material contains Cu_2O crystalline phase, which is due to the small diameter of the CuM nanoparticles, making the material prone to oxidation. However, some information can still be obtained, such as the (200) crystal plane of Cr0 at 64.58°in CuCr, and the absence of the (110) crystal plane at 44.39°. The XRD peaks in CuNi are very close but distinguishable, with no interference between the Cu and Ni peaks (Figure S1). Combined transmission electron microscopy and X-ray powder diffraction characterizations provide preliminary validation for the successful preparation of CuM nanoparticles.

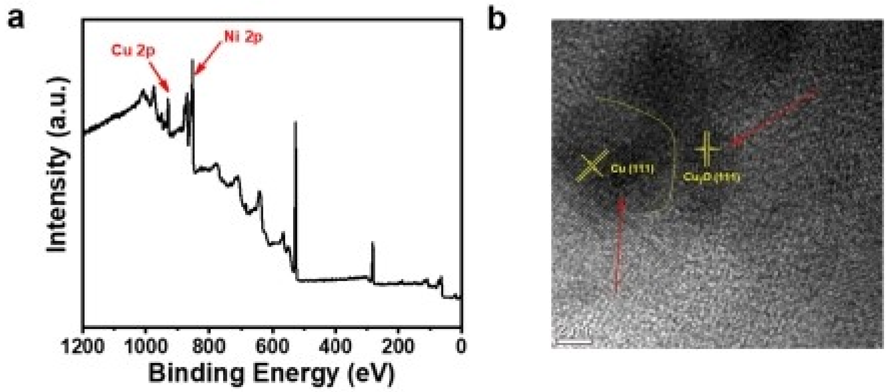

Fig. S3. (a) XPS survey spectrum of Cu-Cu_2O/$Ni(OH)_2$. (b) The interface of Cu-Cu_2O by HR-TEM.

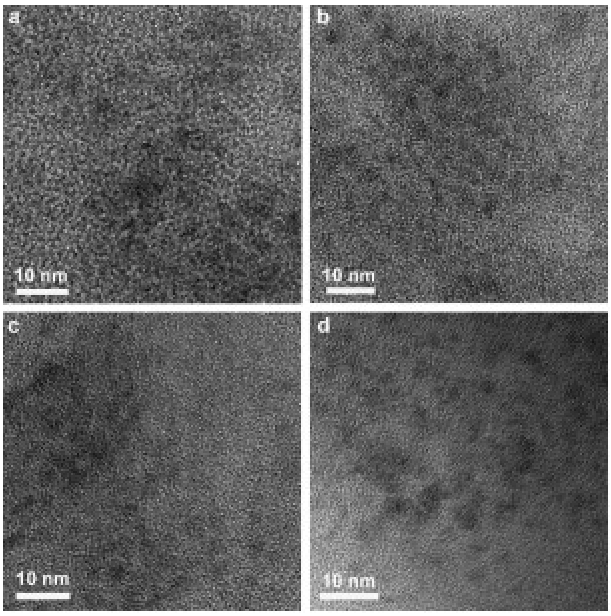

Fig. S4. HR-TEM images of Cu-$Cu_2O/Ni(OH)_2$ by different ultrasound times (5 min、10 min、30 min and 60 min are attributed to a, b, c, and d respectively).

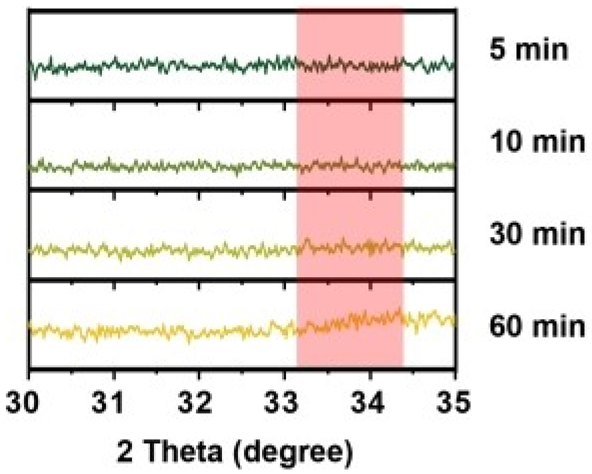

Fig. S5. XRD patterns of CuNi nanoparticles subjected to ultrasound at different times. The transparent pink rectangular region is the $Ni(OH)_2$ (100) in Fig. S6. Although there is a slight enhancement of the signal intensity at the x-ray diffraction angle (2θ) from 30 to 35° for Cu-Cu_2O/$Ni(OH)_2$, it is still considered to be baseline noise, so we assume that no $Ni(OH)_2$ peak was observed at around 33.06°.

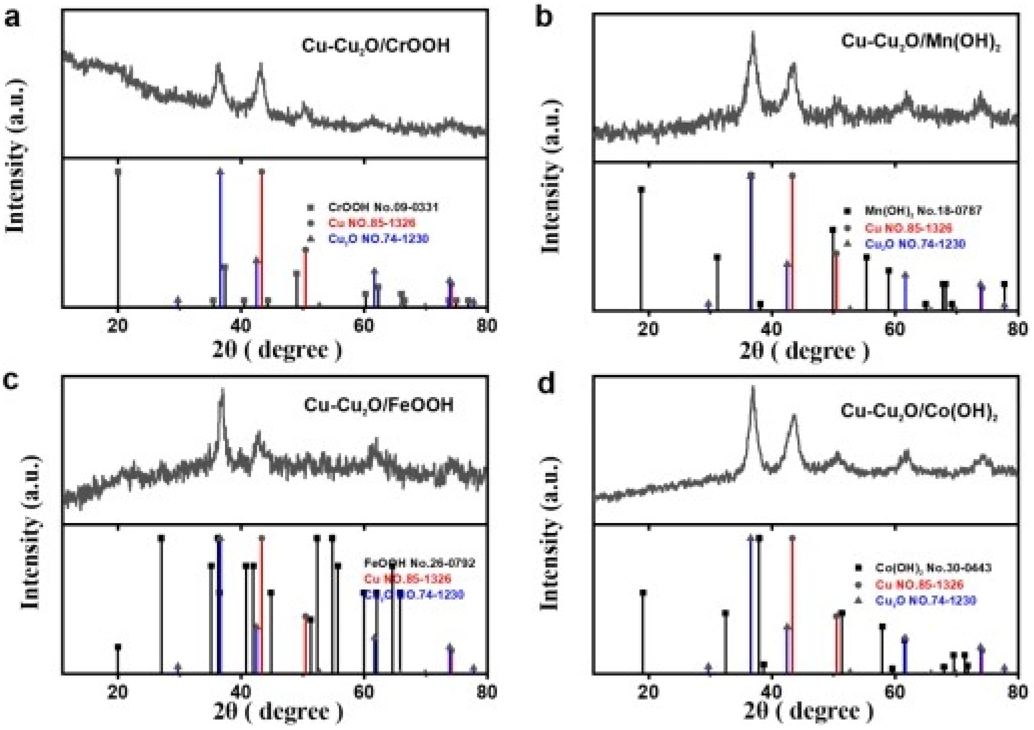

Fig. S6. Corresponding XRD pattern of Cu-$Cu_2O/MO_x(OH)_y$. This section shows the characteristic diffraction peaks of face-centered cubic Cu (111) and (200) at 43.3° and 50.4°, respectively, as well as simple cubic Cu_2O (111) and (220) at 36.6° and 61.6°. The coexistence of Cu and Cu_2O suggests a synergistic interfacial effect that provides active sites for electron transfer and catalysis. In Cu-Cu_2O/CrOOH, Cr doping does not significantly alter the Cu crystalline phase but introduces weak diffraction peaks in the low-angle region, likely associated with the amorphous nature of CrOOH. For Cu-$Cu_2O/Mn(OH)_2$, Mn, Fe, and Co doping markedly enhance the Cu_2O diffraction peak intensity, indicating the promotion of Cu_2O formation and the establishment of Cu-oxide interfaces. In Cu-Cu_2O/FeOOH, Fe doping induces slight lattice stress, leading to a shift of Cu_2O diffraction peaks to higher angles, likely due to the incorporation of Fe atoms into the Cu_2O lattice. Furthermore, no diffraction peaks corresponding to metallic Cr, Mn, Fe, or Co were detected, suggesting the absence of pure Cr, Mn, Fe, or Co phases in the Cu-$Cu_2O/MO_x(OH)_y$ materials. The shift of Cu_2O diffraction peaks imply that dopants regulate the Cu-oxide interfacial structure through electronic effects and lattice strain, optimizing catalytic activity. Notably, the peak intensity and position of the Cu-$Cu_2O/Co(OH)_2$ sample indicate superior lattice matching, explaining its outstanding performance in electrocatalysis.

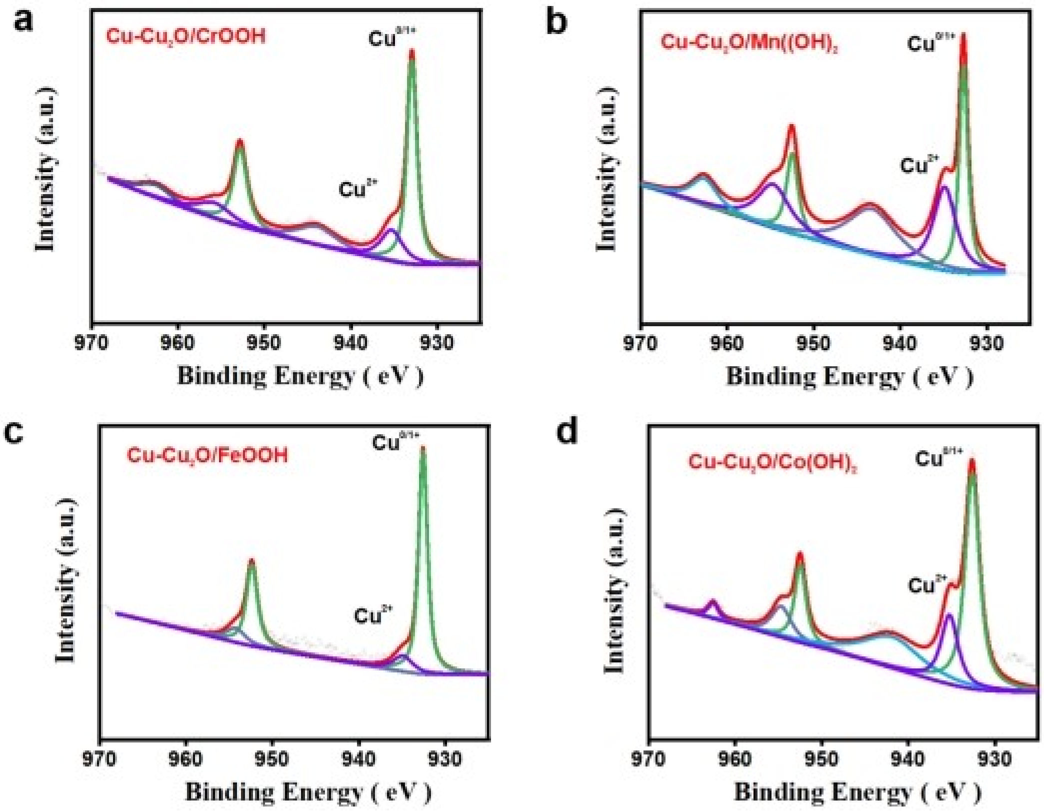

Fig. S7. High-resolution XPS spectra of Cu-$Cu_2O/MO_x(OH)_y$ for Cu2p. The XPS spectra of Cu2p analysis revealed the presence of Cu^0, Cu^{1+}, and Cu^{2+} species in the Cu2p spectra of all materials, with Cu^0 and Cu^{1+} being the dominant states, while Cu^{2+} appeared as weaker satellite peaks. Notably, Cr, Mn, and Co-doped systems exhibited pronounced Cu^{2+} satellite peaks, indicating an increased proportion of higher oxidation states of Cu. This enhancement may be attributed to electronic transfer effects or alterations in the redox properties induced by these dopants. In contrast, the Fe-doped system showed no detectable Cu^{2+} satellite peaks, suggesting that Cu predominantly existed in the Cu^0 and Cu^{1+} states, with a significantly reduced Cu^{2+} content. This phenomenon could be linked to electron transfer effects or redox buffering associated with Fe doping, which suppress further oxidation of Cu. Comparatively, the higher Cu^{2+} content in the Cr, Mn, and Co-doped systems suggests potential advantages for oxidation reactions. Conversely, the Fe-doped system, with its reduced Cu^{2+} proportion, may exhibit greater potential for reductive catalytic processes. However, it is important to note that this does not necessarily imply superior catalytic performance in nitrate reduction, as such reactions are influenced by a combination of factors.

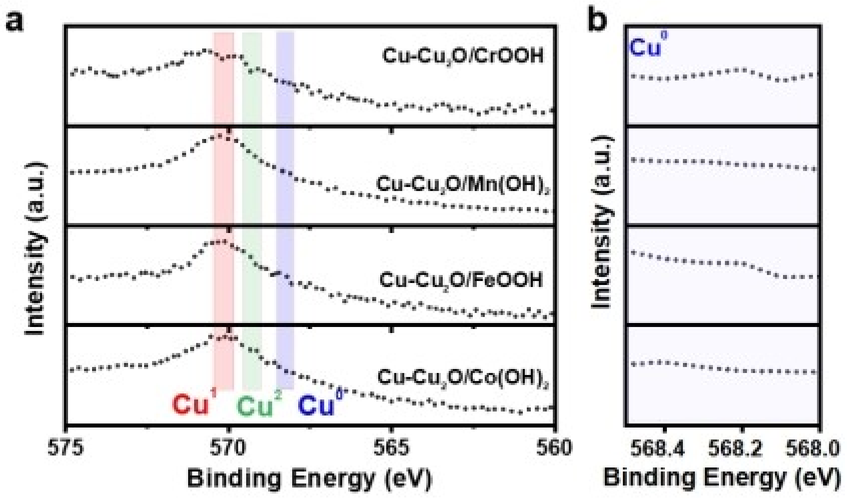

Fig. S8. (a) High-resolution XPS spectra of Cu-$Cu_2O/MO_x(OH)_y$ for Cu LMM. (b) Part enlargement of the blue area in the left figure. To distinguish between Cu^0 and Cu^+, Cu Auger electron spectroscopy (AES) was utilized. In the Cu LMM Auger spectrum (Figure a), the characteristic peaks at approximately 568.2 eV, 568.9 eV, and 570.2 eV correspond to Cu^0, Cu^{2+}, and Cu^+, respectively. Notably, for the CrOOH and FeOOH systems, the Cu^0 peak is located at 568.2 eV, whereas for the $Mn(OH)_2$ and $Co(OH)_2$ systems, the Cu^0 peak shifts to 568.4 eV. The variation in the Cu^0 peak position from 568.2 eV to 568.4 eV indicates a strong sensitivity of the Cu electronic state to the chemical and electronic properties of the dopant elements. In the CrOOH and FeOOH systems, Cu^0 exhibits a lower binding energy, representing an electron-rich state that may facilitate the generation of active hydrogen. Conversely, in the $Mn(OH)_2$ and $Co(OH)_2$ systems, Cu^0 shows a higher binding energy, reflecting an electron-deficient state that may enhance nitrate adsorption. Therefore, this fine-tuning of the electronic state of Cu^0 could significantly influence the adsorption and activation of nitrate intermediates during the catalytic reaction.

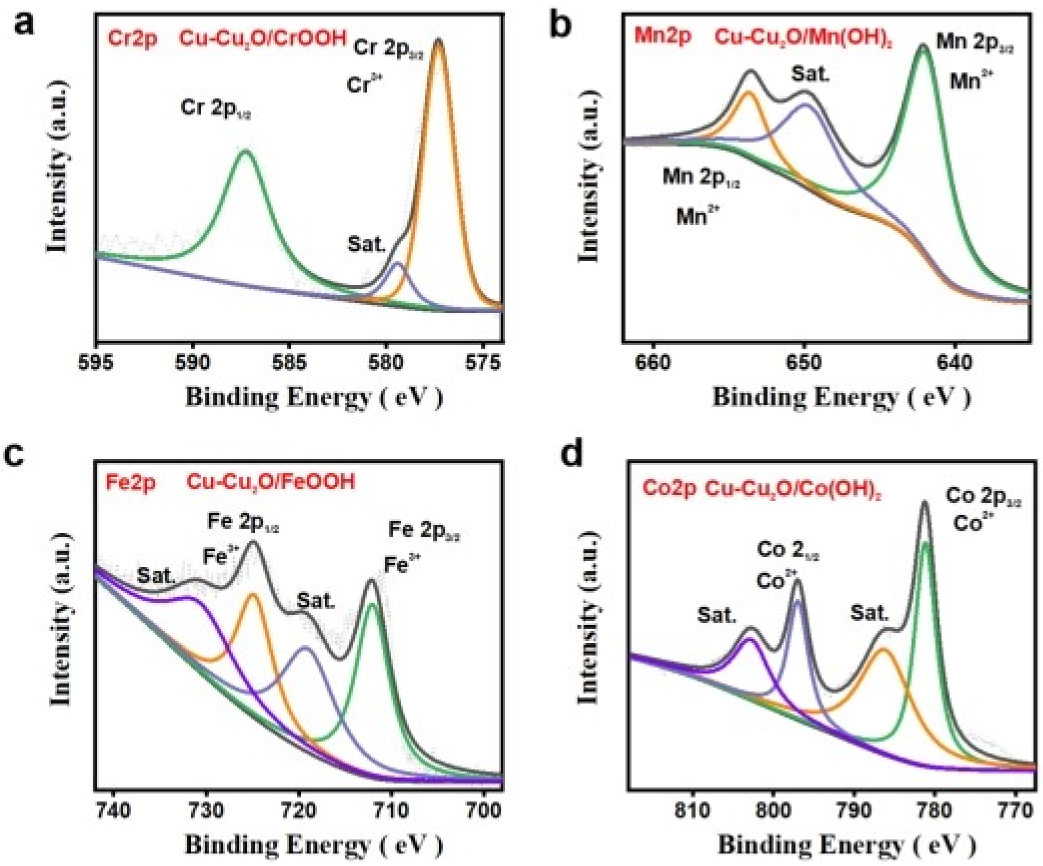

Fig. S9. High-resolution XPS spectra of Cu-$Cu_2O/MO_x(OH)_y$ for M2p. The analysis of XPS M2p spectra (Cr2p, Mn2p, Fe2p, and Co2p) combined with Cu LMM spectra provides an in-depth understanding of the electronic structure and the interactions between copper and dopant elements (Cr, Mn, Fe, and Co) in bimetallic systems. In the M2p spectra, significant binding energy shifts and variations in satellite peaks are observed, reflecting changes in the oxidation states and local chemical environments of the dopant elements. For instance, Cr and Fe exhibit higher oxidation states (M^{3+}), suggesting their role as electron acceptors. In contrast, Mn and Co display lower oxidation states (M^{2+}), which may enhance electron transfer and stabilize reaction intermediates during catalytic processes, thereby facilitating reduction pathways. The combined M2p and Cu LMM spectra underscore the pivotal role of dopant elements in modulating the electronic environment of copper, ultimately influencing its catalytic performance in nitrate reduction reactions.

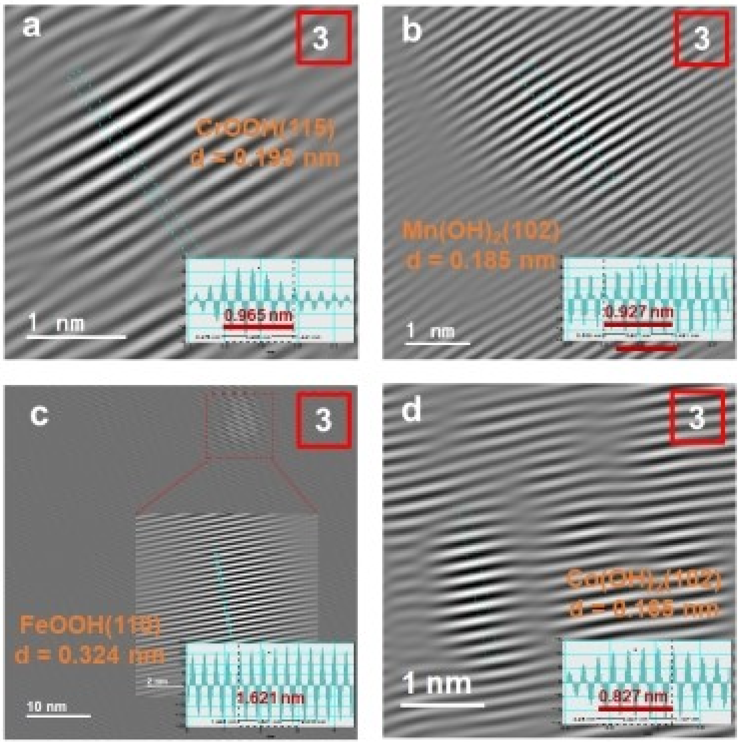

Fig. S10. Facet type and facet spacing of $MO_x(OH)_y$ of Cu-Cu_2O/$MO_x(OH)_y$. Marker 3 in the red box refers to the square box area marked 3 in the second column of Figure 4. High-resolution lattice images reveal distinct interplanar spacings, highlighting structural variations and their potential impact on catalytic performance. CrOOH (115) exhibits an interplanar spacing of 0.193 nm, indicating a compact layered structure that may enhance surface-active site availability. $Mn(OH)_2$ (102) features a slightly smaller spacing of 0.185 nm, suggesting stronger electronic interactions. In contrast, FeOOH (110) has a significantly larger spacing of 0.324 nm, reflecting a loose layered structure that may provide more reaction space but longer electron transfer pathways. $Co(OH)_2$ (102), with the smallest spacing of 0.165 nm, demonstrates the highest lattice compactness, likely improving electron transfer and reactant adsorption. The tight lattices of $Co(OH)_2$ and $Mn(OH)_2$ may impose substantial strain on the Cu/Cu_2O system, modifying its electronic structure to enhance catalytic activity, whereas the looser FeOOH lattice may release strain but reduce electron density, potentially limiting catalytic efficiency.

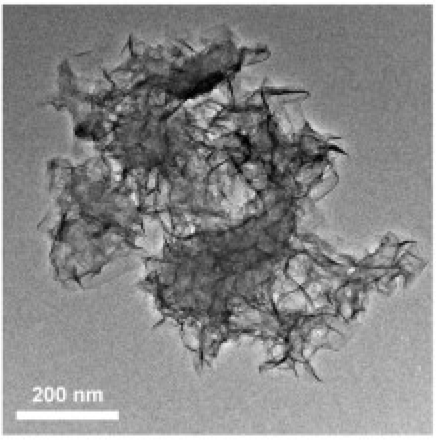

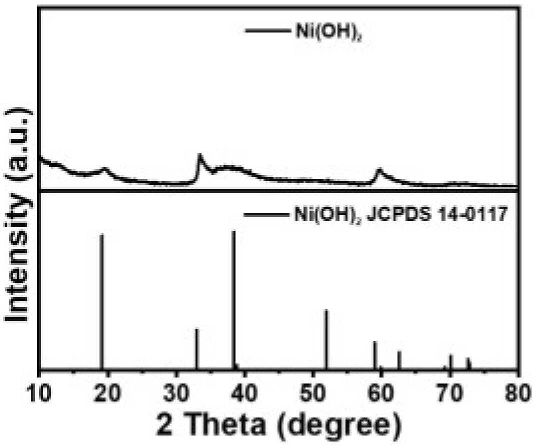

Fig. S11. TEM images and XRD patterns of $Ni(OH)_2$ nanosheets. The low and broad peak intensity and the small shift in the x-ray diffraction angle indicate that $Ni(OH)_2$ is poorly crystallized.

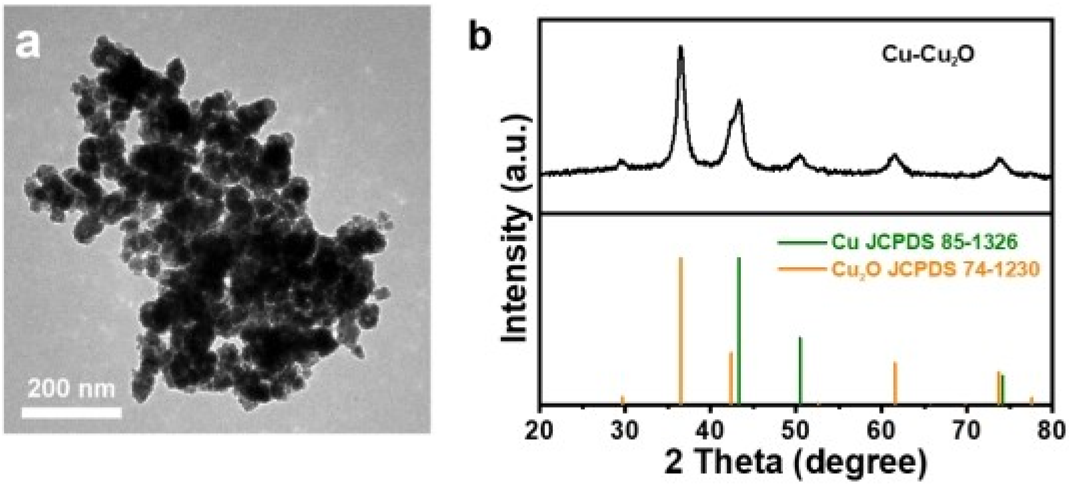

Fig. S12. TEM images and XRD patterns of Cu-Cu_2O nanoparticles. The diffraction peaks at 43.30, 50.43, and 74.13° can be assigned to the (111), (200), and (220) phases of the Cu, respectively.

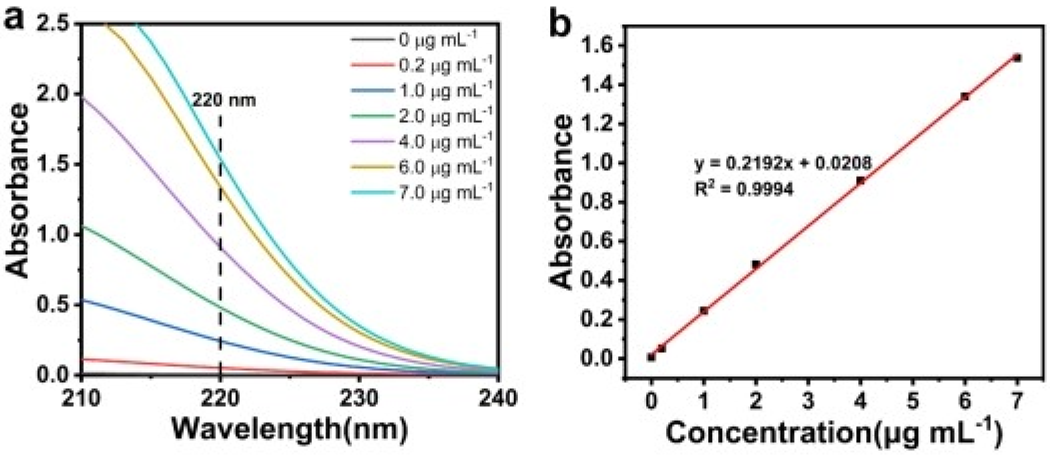

Fig. S13. Standard curves of NO_3^- quantification by coloration method. (a) UV absorption spectra of different concentrations of nitrate-N (b) and the corresponding standard curve. Deionized water was used as a background solution. The calibration curve showed good linearity.

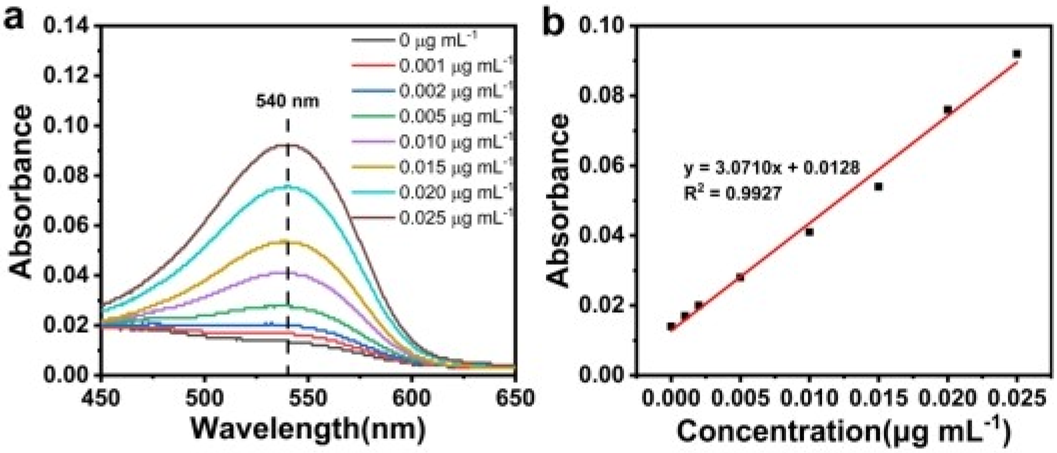

Fig. S14. Standard curves of NO_2^- quantification by coloration method. (a) UV-vis absorption spectroscopy of diazotization spectrophotometry with different concentrations of nitrite-N (b) and the corresponding standard curve. Sodium nitrite was used as source of nitrite-N for the preparation of standard curves. Deionized water was used as a background solution. The calibration curve showed good linearity.

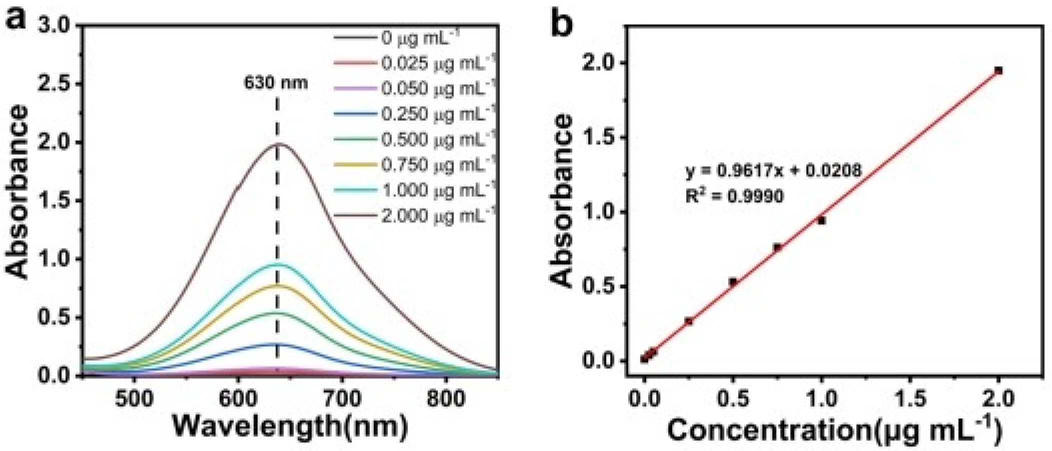

Fig. S15. Standard curves of NH_3 quantification by coloration method (a) UV-vis absorption spectroscopy of indophenol blue spectrophotometry with different concentrations of ammonia-N (b) and the corresponding standard curve. Ammonium chloride was used as source of ammonia-N for the preparation of standard curves. Deionized water was used as a background solution. The calibration curve showed good linearity.

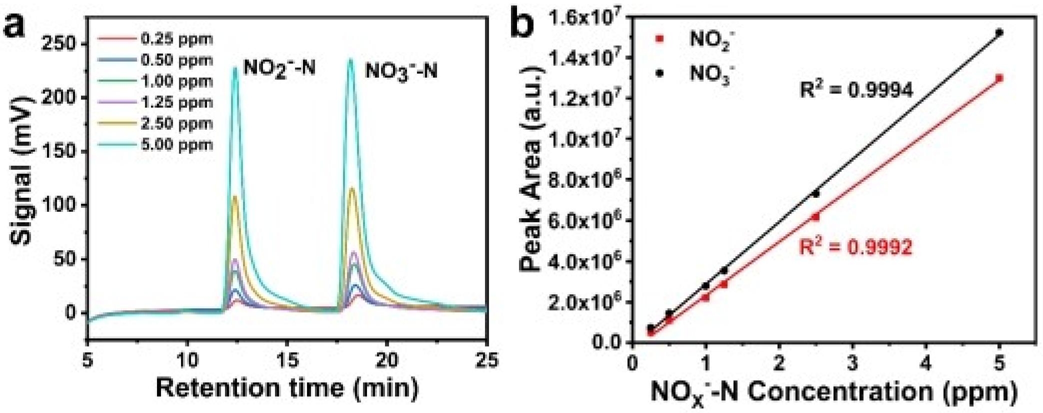

Fig. S16. Calibrations with NO_3^-/NO_2^- standard solutions. (a) IC graphs with standard $NaNO_2$ and $NaNO_3$ solutions. The retention time is 12.1 or 17.8 min for NO_2^- or NO_3^-, respectively. (b) Calibration curves for NO_2^- and NO_3^-. The calibration curves both showed good linearity.

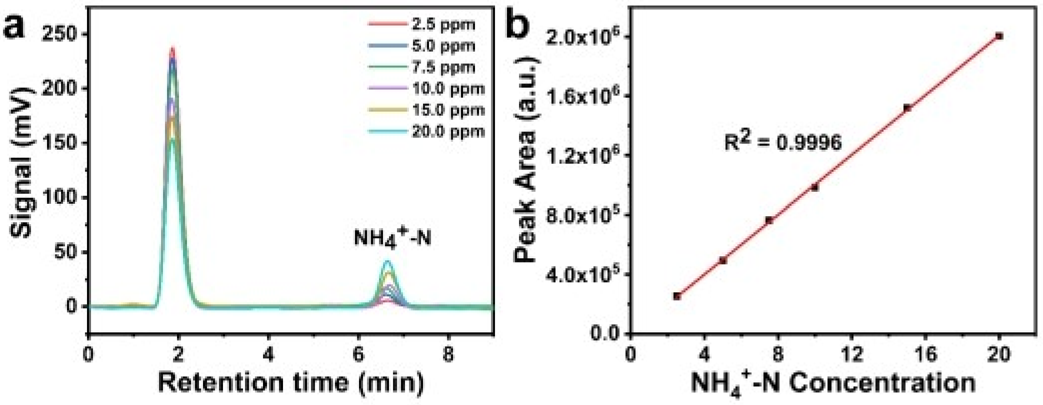

Fig. S17. Calibrations with NH_4^+ standard solutions. (a) IC graphs with standard NH_4Cl solutions. The retention time is 6.7 min for NH_4^+. (b) Calibration curve for NH_4^+. The calibration curve showed good linearity.

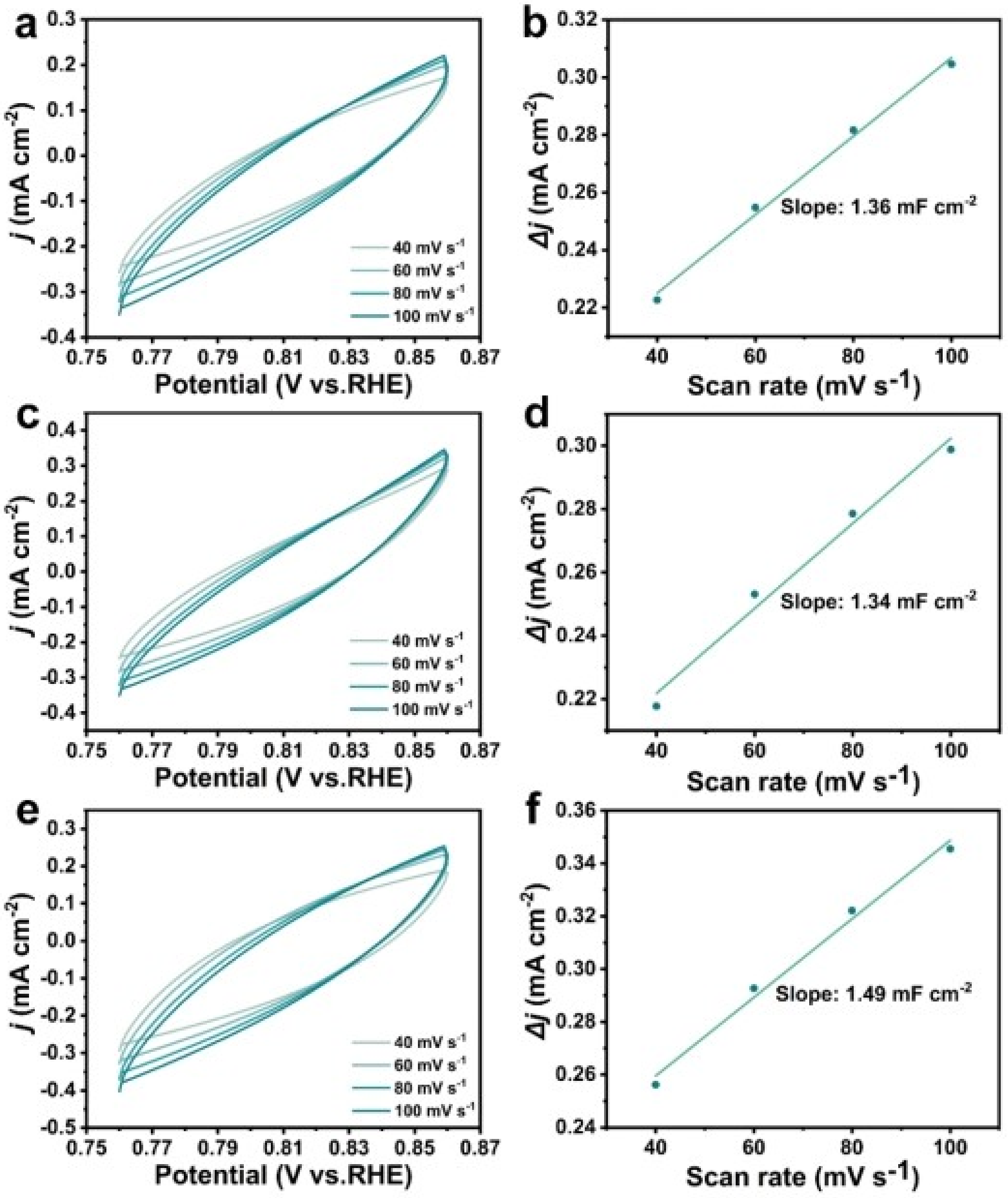

Fig. S18. ECSA measurements of catalyst. CV curves of (a) $Ni(OH)_2$, (c) Cu-Cu_2O (e) and Cu-Cu_2O/$Ni(OH)_2$ with various scan rates from 40 to 100 mV s^{-1}. Fitting lines of the current density versus the different scan rates for (b) $Ni(OH)_2$, (d) Cu-Cu_2O, and (f) Cu-Cu_2O/$Ni(OH)_2$ catalyst.

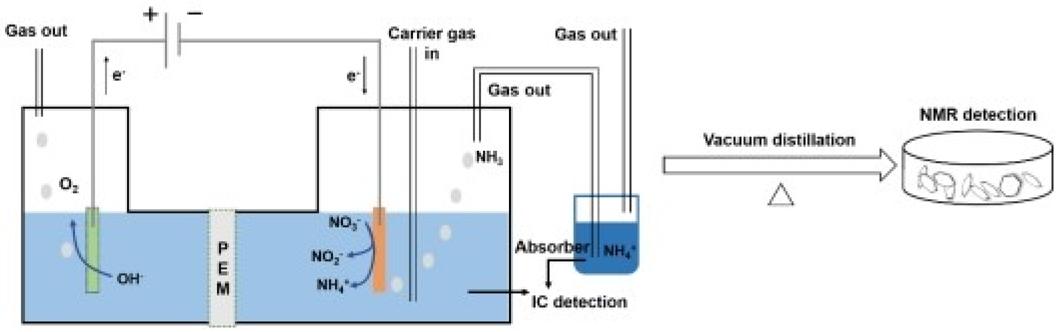

Fig. S19. NO_3^-RR synthetic ammonia collection process. The generated NH_3 was collected in the HCl solution. Next, all of the sample solution was rotary evaporated to remove water and excess HCl, a white powder sample was obtained. Then, the power sample was mixed with 10 mg maleic acid ($C_4H_4O_4$, internal standard) and dissolved in 0.6 mL DMSO-d6. Finally, the prepared mixture was tested by a JNM-ECS 400M spectrometer at ambient conditions and the NH_3 product peaks were analyzed.

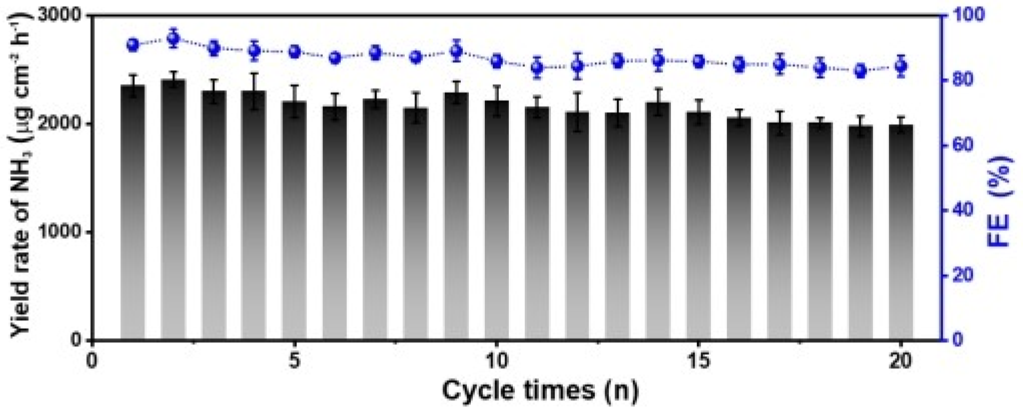

Fig. S20. The stability tests over Cu-Cu_2O/$Ni(OH)_2$. The black column represents the yield rate. The blue ball is FE of ammonia.

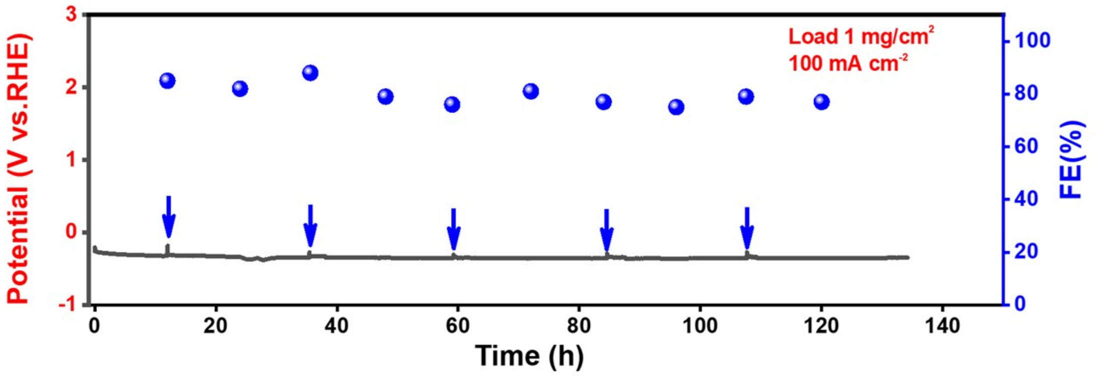

Fig. S21. The long-term electrocatalytic stability of eNITRR on Cu-$Cu_2O/Ni(OH)_2$ was evaluated in an H-type cell at a current density of 100 mA cm^{-2}. The blue arrows indicate the replenishment of fresh electrolytes. The results show that the catalyst maintains approximately 91% of its initial current density and high FE over 130 hours of continuous operation at 100 mA·cm^{-2}. Compared to the data at 20 mA·cm^{-2}, the catalyst exhibits excellent structural integrity and catalytic robustness even under these more rigorous conditions. This further confirms the superior durability of our redox-potential-mediated 0D/2D heterojunction architecture for practical nitrate-to-ammonia conversion.

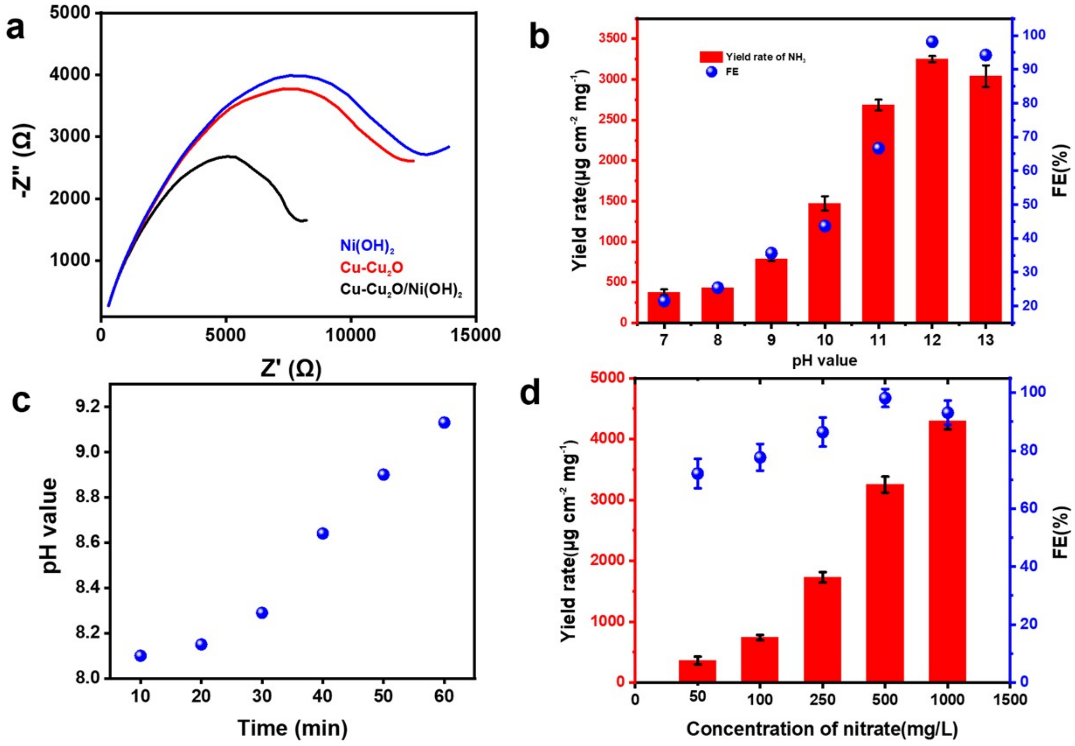

Fig. S22. (a) Nyquist plots of Cu–Cu_2O, $Ni(OH)_2$, and Cu–$Cu_2O/Ni(OH)_2$ heterojunctions obtained at in 0.1 M NaOH + 500mg/L $NaNO_3$. (b) NH_3 Faradaic efficiency (FE) and yield rate of Cu–$Cu_2O/Ni(OH)_2$ from neutral to alkaline electrolytes containing. (c) Real-time local pH changes at the Cu–$Cu_2O/Ni(OH)_2$ electrode surface over different reaction times during eNITRR at -0.01 vs.RHE. (d) FE and yield rate of Cu–$Cu_2O/Ni(OH)_2$ in 0.1 M NaOH with various $NaNO_3$ concentrations ranging from 50 to 1000 mg/L.

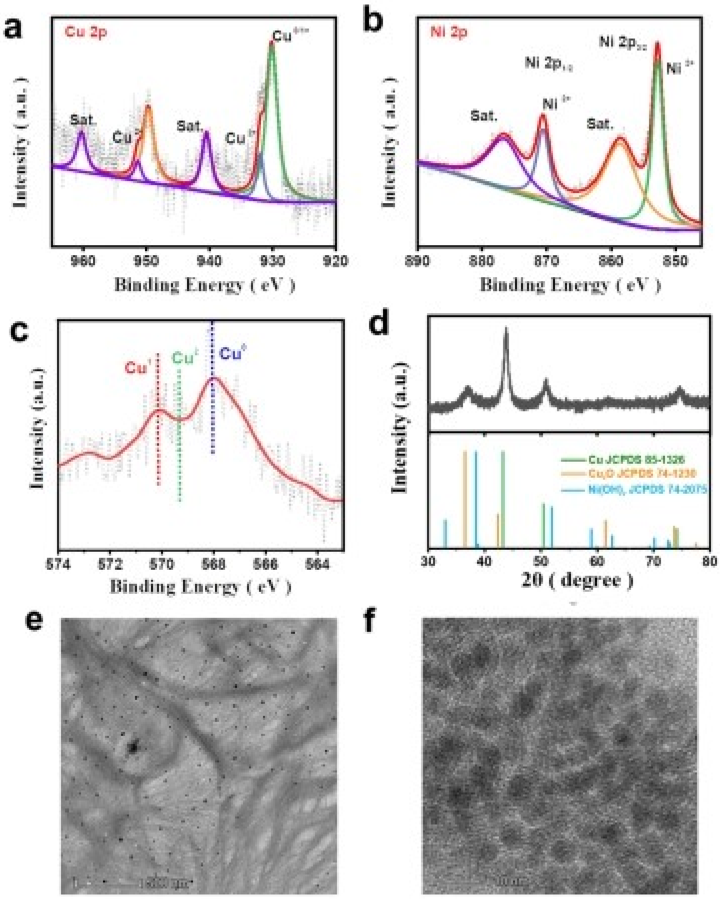

Fig. S23. Characterization after long-term stability test. (a) High-resolution XPS of Cu 2p. (b) High-resolution XPS of Ni 2p. (c) Cu LMM of Cu-Cu_2O/$Ni(OH)_2$. (d) Corresponding XRD pattern of Cu-Cu_2O/$Ni(OH)_2$. (e) TEM image of Cu-Cu_2O/$Ni(OH)_2$. (f) The high-resolution TEM image of Cu-Cu_2O/$Ni(OH)_2$.

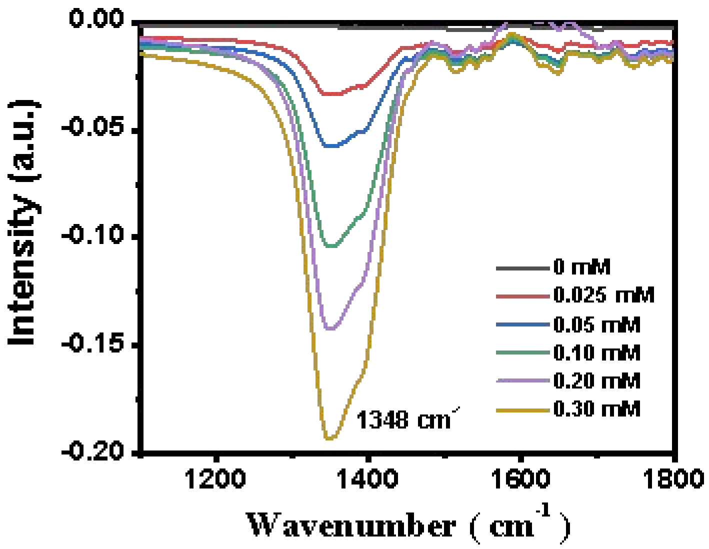

Fig. S24. Ex-situ infrared spectra of nitrate standard samples at varying concentrations.

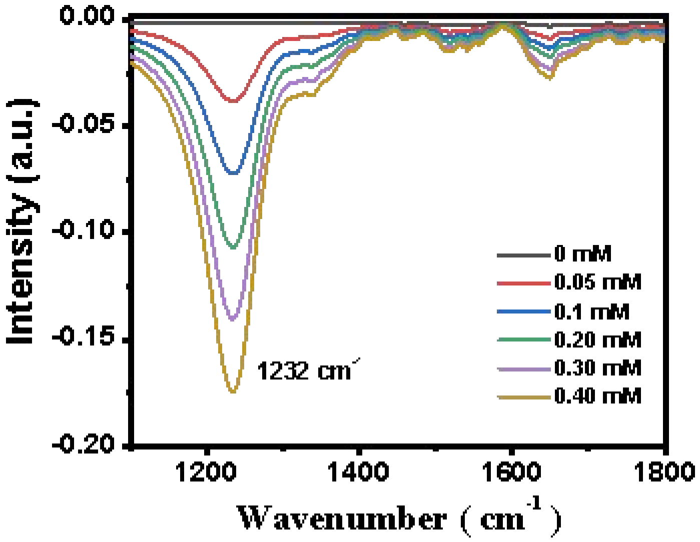

Fig. S25. Ex-situ infrared spectra of nitrite standard samples at varying concentrations.

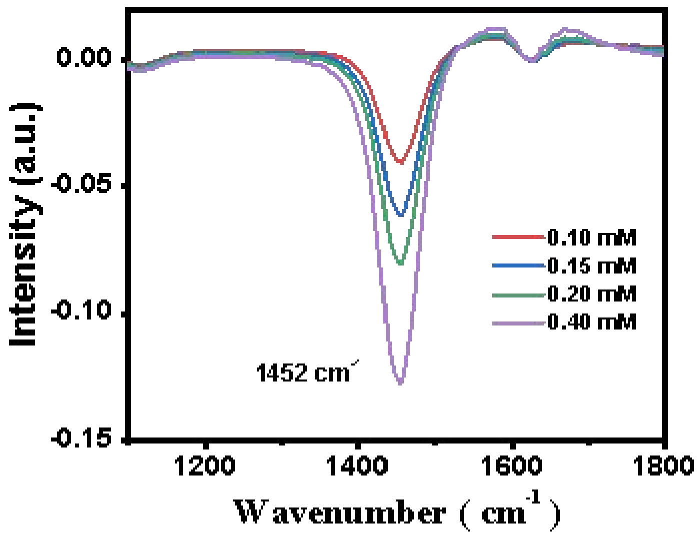

Fig. S26. Ex-situ infrared spectra of $^{15}NH_4^+$ standard samples at varying concentrations.

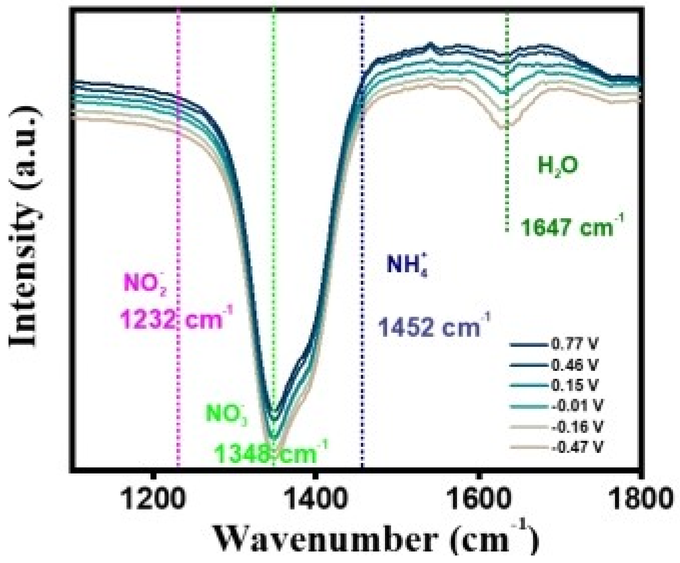

Fig. S27. In situ infrared spectra of $Ni(OH)_2$ – catalyzed with 0.5 mol/L NO_3^- at different potentials.

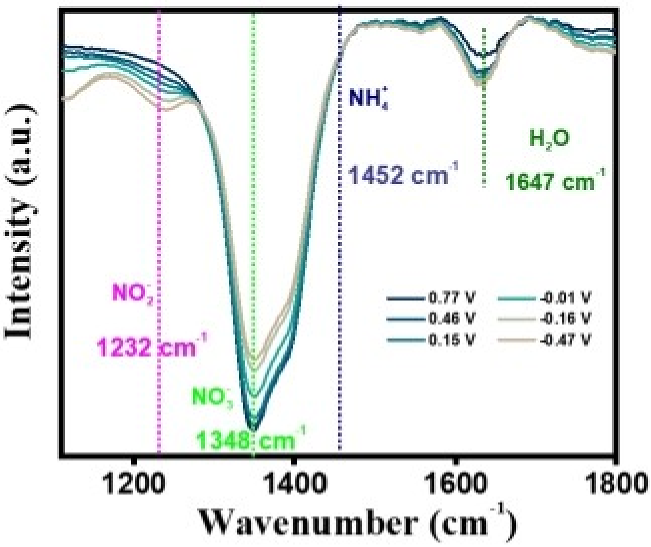

Fig. S28. In situ infrared spectra of Cu-Cu_2O – catalyzed with 0.5 mol/L with NO_3^- at different potentials.

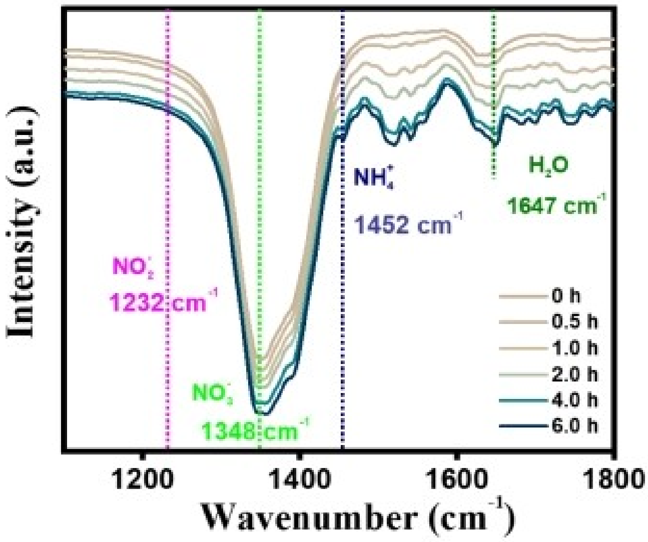

Fig. S29. Isotopic labeling experiments time-resolved in-situ infrared spectra FTIR spectrum. (0.05 mol/L $^{15}NO_3^-$ as N source, pH =13). In the presence of $^{15}NO_3^-$, when the time was varied from 0 h to 6h, clear peaks were observed for Cu-Cu_2O/$Ni(OH)_2$ at 1348 cm^{-1}, 1452 cm^{-1}, and 1647 cm^{-1}, corresponding to the N-O stretching vibration of NO_3^-, the N-H bending vibration of NH_4^+, and the O-H bending vibration of H_2O, respectively**.**

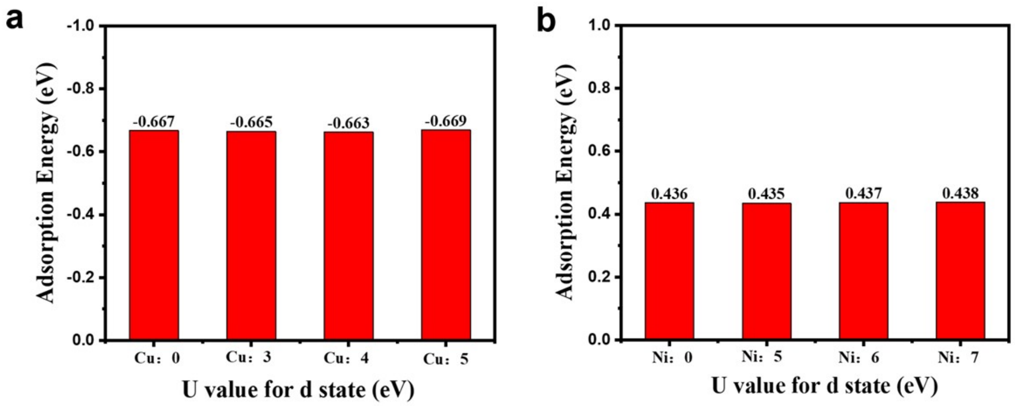

Fig. S30. U value for d state-NO_3^- of (a) Cu-Cu_2O and (b) $Ni(OH)_2$**.** When the U values for both metals are set to zero (i.e., without strong-correlation correction), the adsorption energy (−0.667 eV) differs by less than 0.01 eV from that obtained using the DFT+U approach, indicating that neglecting the strong-correlation correction does not introduce significant errors. Within a reasonable range of U values (Cu: 3–5 eV; Ni: 5–7 eV), the variation in adsorption energy remains negligible (< 0.01 eV), demonstrating that the reaction energetics trends discussed here are highly insensitive to the specific choice of U parameters.

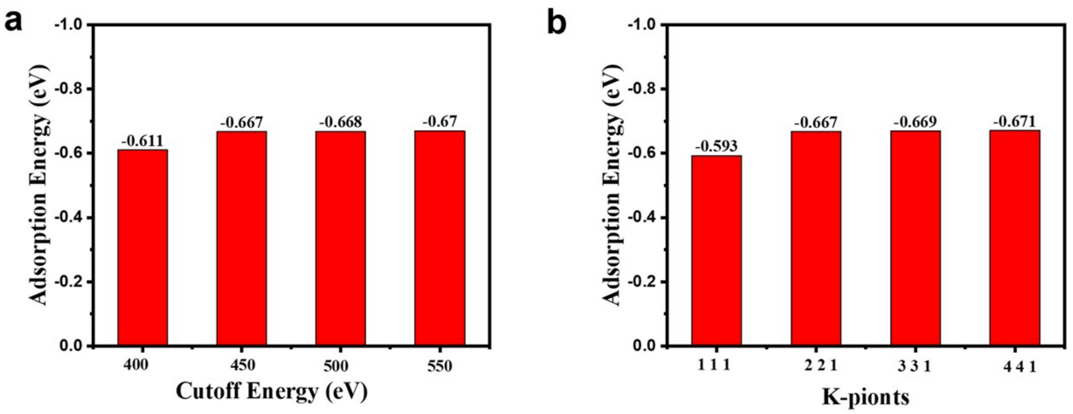

Fig. S31. K-point and plane-wave cutoff energy convergence tests. Sub-figures showing the adsorption energy of $*NO_3$ on the $Cu\text{-}Cu_2O$ surface as a function of (a) cutoff energy and (b) k-point mesh density. The chosen parameters (2×2×1, 450 eV) are marked, showing energy variations within 0.05 eV.

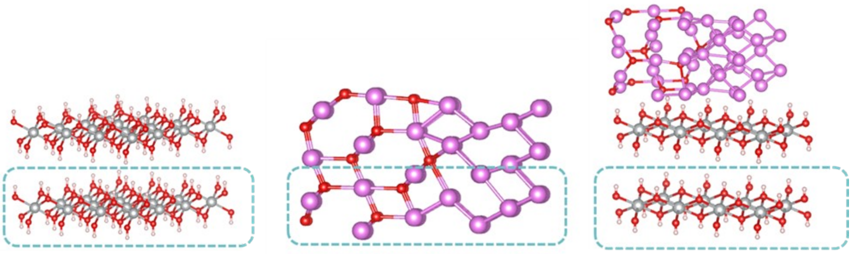

Fig. S32. Schematic illustration of the fixed layers in the slab model of $Ni(OH)_2$, Cu-Cu_2O, Cu-Cu_2O/$Ni(OH)_2$, respectively. During structural optimization, the atoms in the bottom layers of each slab model (indicated by the dashed lines) were fixed to mimic a semi-infinite bulk substrate, while the top layers and all adsorbates were fully relaxed.

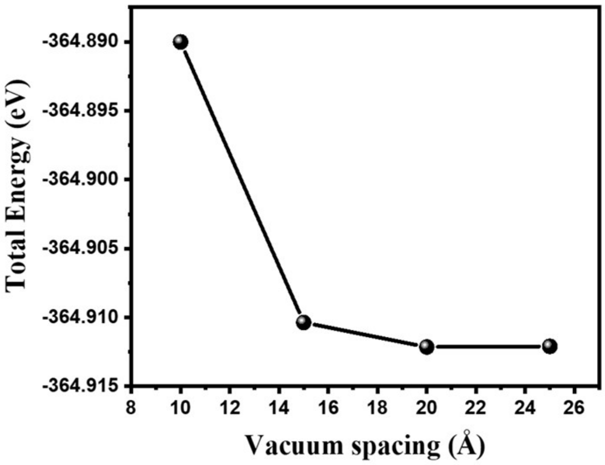

Fig. S33. Convergence of the total energy with respect to vacuum layer thickness. When the vacuum layer thickness reaches 15 Å, the variation in total energy is less than 1 eV, confirming that this thickness is sufficient.

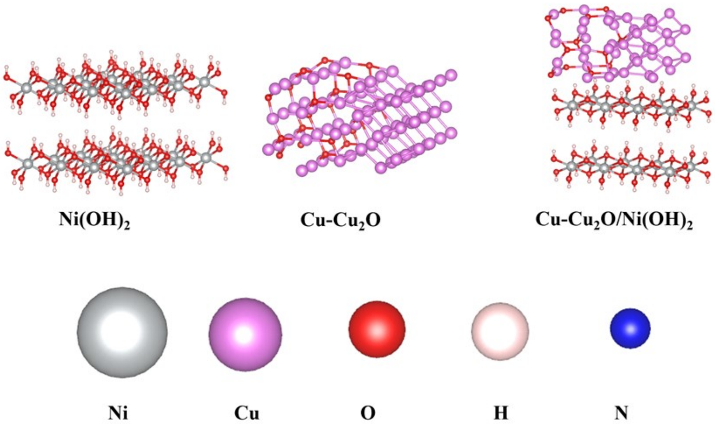

Fig. S34. The crystal structure diagram of $Ni(OH)_2$, $Cu\text{-}Cu_2O$, $Cu\text{-}Cu_2O/Ni(OH)_2$, respectively.

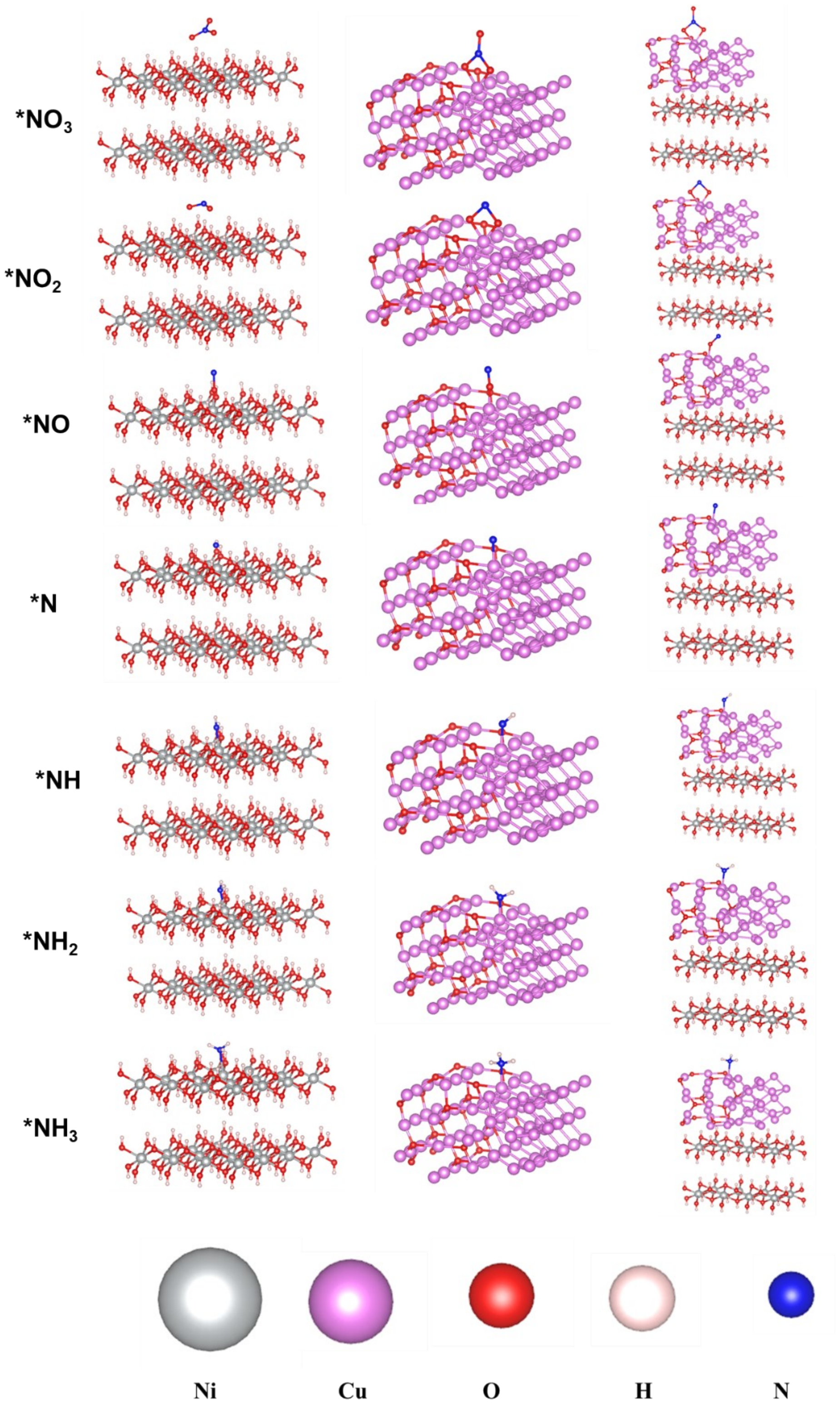

Fig. S35. Modeling of different intermediates in eNITRR processes over Cu-Cu_2O, $Ni(OH)_2$, and Cu-Cu_2O/$Ni(OH)_2$.

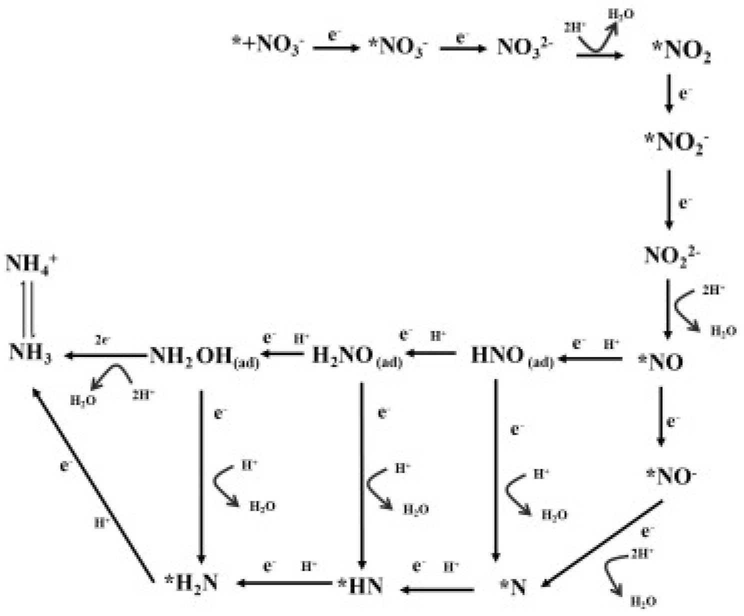

Fig. S36. Pathways of eNITRR. Possible reaction pathways for eNITRR over Cu-$Cu_2O/Ni(OH)_2$.

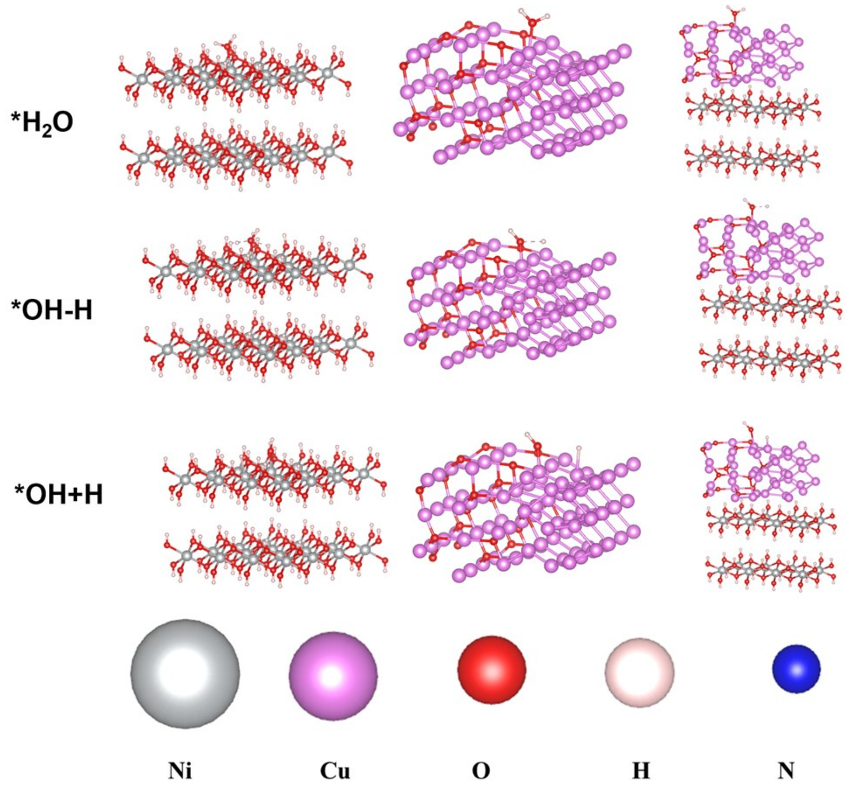

Fig. S37. Modeling of different intermediates in $Ni(OH)_2$, Cu-Cu_2O, and Cu-Cu_2O/$Ni(OH)_2$ during the water activation process.

References

1. Kresse, G.; Furthmüller, J. Efficiency of Ab-Initio Total Energy Calculations for Metals and Semiconductors Using a Plane-Wave Basis Set. *Comput. Mater. Sci*. 1996, **6**, 15−50.
2. Kresse, G.; Furthmüller, J. Efficient Iterative Schemes for Ab Initio Total-Energy Calculations Using a Plane-Wave Basis Set. *Phys. Rev. B* 1996, **54**, 11169−11186.
3. Perdew, J. P.; Burke, K.; Ernzerhof, M. Generalized Gradient Approximation Made Simple. *Phys. Rev. Lett.* 1996, **77**, 3865−3868.
4. Kresse, G.; Joubert, D. From Ultrasoft Pseudopotentials to the Projector Augmented-Wave Method. *Phys. Rev.* B 1999, **59**, 1758-1775.
5. Blöchl, P. E. Projector Augmented-Wave Method. *Phys. Rev.* B 1994, **50**, 17953−17979.
6. M. Yang, Z. Wang, Y. Liu, S. Zhao, J. Zhao and Q. Cai, *Applied Surface Scienc*e, 2023, **620**, 156825.
7. Z. Niu, S. Fan, X. Li, J. Duan and A. Chen, *Applied Catalysis B: Environmental*, 2023, **322**, 122095.
8. W. J. Sun, H. Q. Ji, L. X. Li, H. Y. Zhang, Z. K. Wang, J. H. He and J. M. Lu, *Angewandte Chemie International Editio*n, 2021, **60**, 22933-22939.
9. Y. Guo, R. Zhang, S. Zhang, Y. Zhao, Q. Yang, Z. Huang, B. Dong and C. Zhi, *Energy & Environmental Science*, 2021, **14**, 3938-3944.
10. S. Zhang, M. Li, J. Li, Q. Song and X. Liu, *Proceedings of the National Academy of Sciences*, 2022, **119**, e2115504119.
11. T. Ren, Z. Yu, H. Yu, K. Deng, Z. Wang, X. Li, H. Wang, L. Wang and Y. Xu, *Applied Catalysis B: Environmenta*l, 2022, **31**8, 121855.
12. Z. X. Ge, T. J. Wang, Y. Ding, S. B. Yin, F. M. Li, P. Chen and Y. Chen, *Advanced Energy Material*s, 2022, **12**, 2202081.
13. F. Zhao, G. Li, Q. Hua, J. Cao, J. Song, L. Gao, T. Ma, X. Ren and A. Liu, *Catalysis Science & Technology*, 2023, **1**3, 5543-5548.
14. Y. Xu, K. Shi, T. Ren, H. Yu, K. Deng, X. Wang, Z. Wang, H. Wang, L. Wang and Y. Xu, *Sma*ll, 2022, **18**, 2203335.
15. L. Zhong, Q. Chen, H. Yin, J. S. Chen, K. Dong, S. Sun, J. Liu, H. Xian and T. Li, *Chemical Communication*s, 2023, **59**, 8973-8976.
16. W. Zhang, S. Zhan, J. Xiao, T. Petit, C. Schlesiger, M. Mellin, J. P. Hofmann, T. Heil, R. Muller, K. Leopold and M. Oschatz, *Advanced Science*, 2023, **1**0, 2302623.
17. G.-F. Chen, Y. Yuan, H. Jiang, S.-Y. Ren, L.-X. Ding, L. Ma, T. Wu, J. Lu and H. Wang, *Nature Energy*, 2020, 5, 605-613.
18. Z.-Y. Wu, M. Karamad, X. Yong, Q. Huang, D. A. Cullen, P. Zhu, C. Xia, Q. Xiao, M. Shakouri, F.-Y. Chen, J. Y. Kim, Y. Xia, K. Heck, Y. Hu, M. S. Wong, Q. Li, I. Gates, S. Siahrostami and H. Wang, *Nature Communication*s, 2021, **1**2, 2870.
